# Squamocin Suppresses Tumor Growth through Triggering an Endoplasmic Reticulum Stress‐Associated Degradation of EZH2/MYC Axis

**DOI:** 10.1002/advs.202413120

**Published:** 2025-01-17

**Authors:** Yin Zhu, Yurui Liu, Xiangtao Wang, Zhifeng Chen, Baojian Chen, Bingxin Hu, Tiane Tang, Haoran Cheng, Xinglong Liu, Yunshan Ning

**Affiliations:** ^1^ School of Laboratory Medicine and Biotechnology Southern Medical University Guangzhou 510515 China; ^2^ Guangdong Provincial Key Laboratory of Immune Regulation and Immunotherapy Guangzhou 510515 China; ^3^ Institute of Medicinal Plant Development Chinese Academy of Medical Sciences & Peking Union Medical College Beijing 100193 China; ^4^ Department of Stomatology Nanfang Hospital Southern Medical University Guangzhou 510515 China; ^5^ Southern Medical University Hospital of Integrated Traditional Chinese and Western Medicine Southern Medical University Guangzhou 510000 China; ^6^ The First Clinical Medical School Southern Medical University Guangzhou 510515 China

**Keywords:** annonaceous acetogenins, endoplasmic reticulum stress response, EZH2/MYC axis, squamocin, ubiquitin‐proteasome degradation system

## Abstract

Despite substantial advances in the antitumor effects of annonaceous acetogenins (ACGs), the absence of a defined biological action mechanism remains a major barrier to their clinical application. Here, it is found that squamocin effectively depletes both EZH2 and MYC in multiple cancer cell lines, including head and neck squamous cell carcinoma, and gastric and colorectal cancer, demonstrating potent efficacy in suppressing these in vivo tumor models. Through the combination of surface plasmon resonance (SPR), differential scanning fluorimetry (DSF), and cellular thermal shift assay (CETSA), heat shock protein 90α (HSP90α) is identified as the direct binding target of squamocin. Mechanistically, squamocin disrupts mitochondrial respiratory Complex I function, reduces ATP production, and impairs HSP90α function, provoking endoplasmic reticulum (ER) stress and the unfolded protein response (UPR). These intrinsic events within tumor cells enhance ER stress‐associated ubiquitylation and degradation by triggering ubiquitin via the E1 activase UBA6, facilitating ubiquitin transferring to E2 conjugate UBE2Z and increasing the activities of E3 ligase FBXW7 to degrade both EZH2 and MYC. The findings elucidate the role of squamocin in the degradation of oncoproteins EZH2 and MYC by triggering an ER stress‐associated UBA6‐UBE2Z‐FBXW7 ubiquitin cascade, providing insights that may accelerate therapeutic development targeting tumors driven by the EZH2/MYC axis.

## Graphical abstract

Squamocin disrupts mitochondrial respiratory Complex I function, reduces ATP production, and impairs HSP90α function, provoking endoplasmic reticulum (ER) stress and the unfolded protein response (UPR) in multiple cancer tumor models cell lines and in vivo tumor models. These intrinsic events within tumor cells enhance ER stress‐associated ubiquitylation and degradation of the EZH2/MYC axis by triggering the UBA6‐UBE2Z‐FBXW7 ubiquitin cascade.

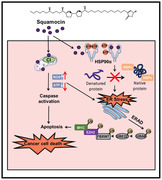

## 1 Introduction

Head and neck squamous cell carcinoma (HNSCC) is an aggressive malignancy characterized by a high proliferation rate, significant recurrence, and a poor prognosis.^[^ ^1^ ^]^ Despite advancements in surgical techniques and chemoradiotherapy over recent decades, therapeutic outcomes remain unsatisfactory, with more than 50% of patients experiencing recurrence or metastasis within three years of treatment, leading to dismal prognoses.^[^ ^2^ ^]^ Given the limited efficacy of systemic cytotoxic chemotherapy in treating recurrent or metastatic (R/M) HNSCC, recent clinical efforts have shifted to immune checkpoint inhibitors (ICIs); but only 15%–20% of patients experience a survival benefit.^[^ ^3^ ^]^ To date, the mechanisms underlying resistance to ICIs in R/M HNSCC remain poorly understood; however, MYC activation has been shown to contribute to ICIs resistance in both R/M HNSCC^[^ ^4^ ^]^ and esophageal squamous cell carcinoma.^[^ ^5^ ^]^ These data suggest that MYC activation may represent a potential mechanism of resistance to ICIs, underscoring the necessity of understanding how MYC activation drives tumorigenesis and highlighting the urgent need for effective strategies targeting MYC‐driven tumors.

MYC, encoded by the *MYC* proto‐oncogene, is dysregulated in up to 70% of all human cancers through mechanisms, including genetic copy‐number gain, aberrant upstream signaling, and altered protein stability,^[^ ^6^ ^]^ and contributes to almost every aspect of tumorigenesis.^[^ ^7^ ^]^ To date, numerous therapeutic agents directly targeting MYC are under development; however, their clinical efficacy has yet to be established, indicating that alternative strategies for targeting MYC may need further exploration. Epigenetic proteins have emerged as critical regulators of MYC at multiple levels to control its activity,^[^ ^6^, ^8^ ^]^ and these druggable proteins represent an attractive strategy for inhibiting oncogenic MYC function.

The histone methyltransferase enhancer of zeste homologue 2 (EZH2), a core subunit of the Polycomb repressive complex 2 (PRC2), functions together with suppressor zeste 12 (SUZ12) and embryonic ectoderm development (EED) to catalyze histone H3 lysine 27 trimethylation (H3K27me3) and maintain transcriptional repression.^[^ ^9^ ^]^ Exquisite EZH2 dependencies have been demonstrated across a range of human tumors, including HNSCC, gastric cancer (GC), and colorectal cancer (CRC), where elevated levels of EZH2 are correlated with dysregulated tumor proliferation,^[^ ^10^ ^]^ metastasis,^[^ ^11^ ^]^ and poor outcome.^[^ ^12^ ^]^ It is noteworthy that recent findings across several tumor types have revealed that EZH2 can also exert its oncogenic effects by promoting the stabilization of MYC as its partner,^[^ ^13^ ^]^ thereby providing a rationale for and examples of therapeutic strategies targeting MYC. However, current inhibitors, including EPZ‐6438, GSK126, and UNC1999,^[^ ^14^ ^]^ often induce slow and/or partial cellular responses,^[^ ^15^ ^]^ which only inhibit the catalytic activity of EZH2 (catalytic inhibitors), may fail to potentiate the degradation of its MYC partner. Therefore, effectively triggering the degradation of both the EZH2 oncoprotein and its MYC partner represents an attractive therapeutic strategy for cancer.

In various human cancers, tumor cells with MYC activation further fuel endoplasmic reticulum (ER) stress and promote persistent activation of the unfolded protein response (UPR).^[^ ^16^ ^]^ This persistent, non‐lethal UPR facilitates tumor cell proliferation, survival, metastasis, chemoresistance, and immune evasion;^[^ ^16^, ^17^ ^]^ conversely, unresolved or extreme UPR can trigger apoptosis in tumor cells.^[^ ^16^, ^18^ ^]^ However, normal cells are not subjected to ER stress, and the UPR pathway is inactive.^[^ ^16^ ^]^ Therefore, inducing an unresolved or lethal UPR in tumor cells, particularly within the context of MYC‐driven tumors, represents a novel therapeutic approach with minimal effects on healthy cells.^[^ ^18^ ^]^

Annonaceous acetogenins (ACGs) are a unique and structurally homogeneous class of polyketides derived from annonaceae plants, demonstrating potent antiproliferative effects across various cancers.^[^ ^19^ ^]^ Squamocin, one of the most active components of ACGs,^[^ ^20^ ^]^ exhibits significant cytotoxicity against a range of cancer cells and chemoresistant malignancies,^[^ ^21^ ^]^ being ≈100 times more effective than adriamycin against MCF‐7 cells.^[^ ^21b^ ^]^ Furthermore, squamocin has been reported to modulate histone H3 phosphorylation levels, induce G1 phase arrest in multiple cancer cell lines,^[^ ^22^ ^]^ and block chronic myeloid leukemia cells in the G2/M phase.^[^ ^23^ ^]^ Additionally, squamocin demonstrates selective cytotoxicity toward S‐phase‐enriched T24 bladder cancer cells,^[^ ^21a^ ^]^ and induces apoptosis in HL‐60 leukemia cells via caspase 3 activation and stress‐activated protein kinase (SAPK/JNK) activation.^[^ ^24^ ^]^ Despite these advances, our understanding of the mechanisms underlying these effects, specifically how squamocin arrests tumor growth remains limited. Moreover, as a cytotoxic agent and an inhibitor of mitochondrial respiratory Complex I that disrupts the ATP production in tumor cells,^[^ ^25^ ^]^ it remains to be determined whether squamocin can trigger a lethal UPR in human carcinoma, particularly in cancers with MYC activation. Finally, the direct molecular targets and associated signaling pathways of squamocin have yet to be elucidated.

To address these knowledge gaps, we investigated the antitumor effects of squamocin in preclinical models and clinical samples from HNSCC, GC, and CRC, where both EZH2 and MYC activation converge. We elucidated the mechanisms by which squamocin induces ER stress, leading to ER‐associated degradation (ERAD) of EZH2 and MYC proteins, ultimately resulting in tumor growth arrest.

## 2 Results

### 2.1 Squamocin Suppresses the Proliferation of HNSCC Cell Lines with High MYC Expression

**Figure 1:**
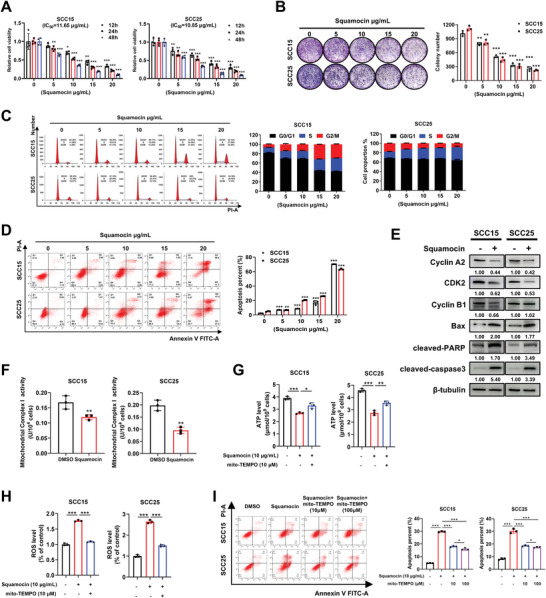
Squamocin suppresses proliferation, arrests the cell cycle, and promotes apoptosis in HNSCC. A) SCC15 and SCC25 cells were treated with squamocin at the indicated concentrations for 12, 24, and 48 h. Cell viabilities were measured by CCK8 (mean ± SEM; *n* = 4, Student's *t*‐test). B) Representative images of the clonogenic assay and the quantification of colonies per well for cells treated with indicated concentrations of squamocin for 10 days (mean ± SEM; *n* = 3, Student's *t*‐test). C, D) Cells were treated with indicated concentrations of squamocin for 24 h for cell cycle analysis by PI staining (C), and for analysis of apoptosis by Annexin V staining (D) (mean ± SEM; *n* = 3, Student's *t*‐test). E) Immunoblotting of indicated proteins in SCC15 and SCC25 cells treated with DMSO or 10 µg mL^−1^ squamocin for 24 h. F) SCC15 and SCC25 cells were treated with 10 µg mL^−1^ squamocin for 24 h, and the mitochondrial respiratory chain Complex I activity was analyzed (mean ± SEM; *n* = 3, Student's *t*‐test). G‐I) SCC15 and SCC25 cells were pretreated with mito‐TEMPO (10 µm) for 6 h, followed by squamocin (10 µg mL^−1^) treatment for 24 h. After treatment, cells were used to complete the following assays: extracellular ATP level analysis (G), intracellular ROS analysis (H), and apoptosis analysis by Annexin V staining (I) (mean ± SEM; *n* = 3, Student's *t*‐test). ^*^ *p* < 0.05, ^**^ *p* < 0.01, and ^***^ *p* < 0.001.

As a major antitumor component of ACGs, squamocin was isolated from the seed of Annona squamosa according to the procedure reported,^[^ ^26^ ^]^ achieving an HPLC purity of 93.13% (Figure S1, Supporting Information). Notably, we found that squamocin exhibited diverse effects on cells with different levels of MYC expression in that it had a high inhibitory on HNSCC cell lines with high levels of MYC (CAL27, FADU, SCC15, and SCC25), and a weak effect on noncancerous cell lines with low MYC expression (NOK and HUVEC) (Figure S2A, Supporting Information). Next, SCC15 and SCC25 were treated with squamocin at 5 to 20 µg mL^−1^ for 12, 24, and 48 h. Consequently, we found an obvious dose‐ and time‐dependent cell viability inhibition by squamocin in two HNSCC cell lines (**Figure** **1**A; Figure S2B, Supporting Information). The half‐maximal inhibitory concentration (IC_50_) was calculated in SCC15 (IC_50_ = 11.65 µg mL^−1^) and SCC25 (IC_50_ = 10.85 µg mL^−1^) (Figure 1A). Squamocin treatment dramatically decreased the number of colonies in SCC15 and SCC25 cells (Figure 1B). Cell cycle analysis showed that 10 µg mL^−1^ squamocin arrested the cell cycle at the S and G2/M phases in SCC15 cells, and only in the S phase in SCC25 cells (Figure 1C). A significant increase in apoptosis was detected in SCC15 and SCC25 cells at 24 h after treatment with 10 µg mL^−1^ squamocin (Figure 1D). Consistently, S‐phase biomarkers Cyclin A2 and CDK2 were downregulated in both SCC15 and SCC25 cells, while G2/M phase biomarker Cyclin B1 was only downregulated in SCC15 cells (Figure 1E). In addition, the protein expression of Bax, activated caspase 3, and PARP was increased by squamocin (Figure 1E). These results revealed that squamocin suppresses cell proliferation, induces cell cycle arrest, and promotes apoptosis in HNSCC cells.

It has been reported that squamocin inhibits mitochondria respiratory Complex I, thereby obstructing mitochondrial oxidative phosphorylation and inducing apoptosis.^[^ ^19a^ ^]^ To investigate whether the pro‐apoptotic activity of squamocin is attributable to its inhibition of mitochondrial respiratory Complex I, we assessed the effects of squamocin on mitochondria function in two HNSCC cell lines. As anticipated, squamocin significantly inhibited the activity of mitochondrial respiratory Complex I with a reduction in ATP production (Figure 1F,G). When these cells were pretreated with the mitochondria‐specific antioxidant mito‐TEMPO, ATP depletion was partially restored (Figure 1G). Furthermore, squamocin elevated reactive oxygen species (ROS) levels in both HNSCC cell lines; whereas ROS accumulation was significantly inhibited when these cells were pretreated with mito‐TEMPO (Figure 1H). These data confirm that squamocin impairs the function of mitochondrial respiratory Complex I in HNSCC cells. However, unexpectedly, flow cytometry revealed that pretreated two HNSCC cell lines with mito‐TEMPO (10 or 100 µm) only partially inhibited squamocin‐induced apoptosis (Figure 1I), suggesting that other mechanisms may mediate the antitumor effects of squamocin.

### 2.2 HSP90α is the Direct Binding Target of Squamocin

**Figure 2:**
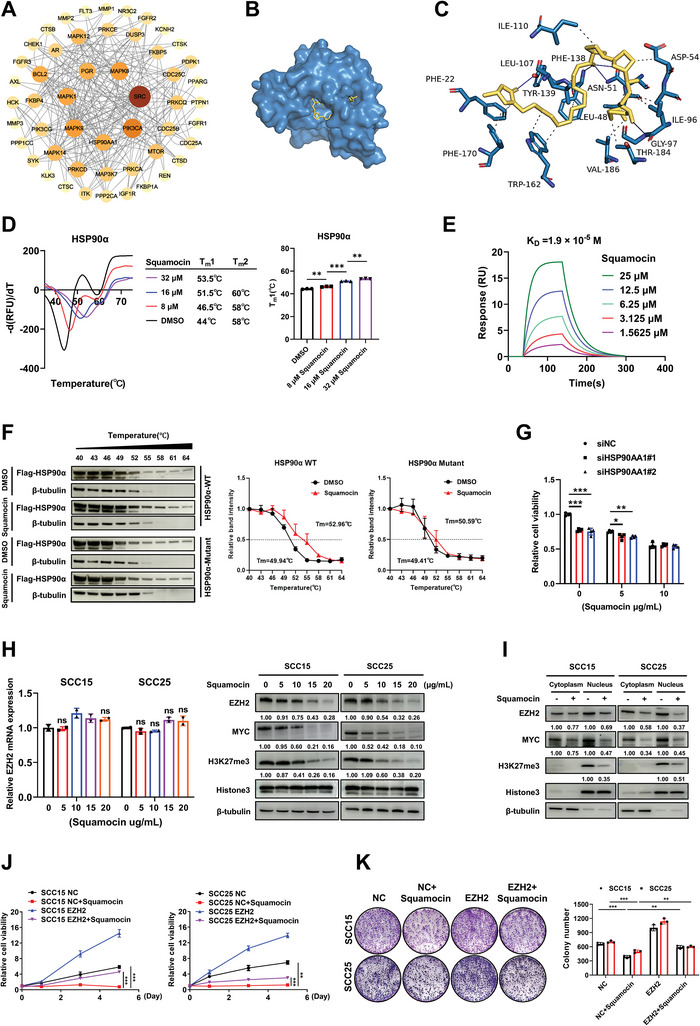
HSP90α is the direct target protein of squamocin. A) Direct target proteins of squamocin were analyzed by target fishing analysis using the SwissTargetPrediction database, followed by protein‐protein interaction analysis of potential targets. B, C) Computational docking showed the binding pattern (B) and interaction diagram (C) of HSP90α and squamocin. D) DSF analysis of squamocin binding to the HSP90α protein (mean ± SEM; *n* = 3, Student's *t*‐test). E) SPR analysis of squamocin binding to the HSP90α protein. F) 293T cells were transfected with plasmids of pcDNA3.1‐3×Flag‐C‐HSP90α WT/HSP90α mutant and the binding between squamocin and HSP90α was analyzed by CETSA. The intensity of the bands for grayscale analysis was referenced to the starting temperature (40 °C), and the curve was fitted using Prism (mean ± SEM; *n* = 3). G) SCC15 cells were transfected with two (#1 and #2) siHSP90AA1 for 48 h followed by treatment with squamocin for 24 h. Cell viabilities were measured by CCK8 (mean ± SEM; *n* = 4, Student's *t*‐test). H) SCC15 and SCC25 cells were treated with corresponding concentrations of squamocin for 24 h, followed by qRT‐PCR analysis for EZH2 mRNA expression (left panel, mean ± SEM; *n* = 2, Student's *t*‐test) and immunoblot analysis of indicated proteins (right panel). I) Western blot analysis of the indicated proteins in the cytoplasmic and nuclear fraction of cells treated with squamocin (10 µg mL^−1^) for 24 h. J) Cell proliferation curves of EZH2‐overexpressing SCC15 and SCC25 cells with squamocin treatment (mean ± SEM; *n* = 4, two‐way ANOVA test). K) Representative images of the clonogenic assay in EZH2‐overexpressing SCC15 and SCC25 cells following squamocin (10 µg mL^−1^) treatment for 10 days and the quantification of clones per well (mean ± SEM; *n* = 3, Student's *t*‐test). ^*^ *p* < 0.05, ^**^ *p* < 0.01, and ^***^ *p* < 0.001.

To address this question, we identified 100 potential protein targets of squamocin with target fishing analysis using the SwissTargetPrediction database (Table S1, Supporting Information). Subsequently, we conducted protein‐protein interaction analysis to investigate key proteins among these targets, employing the STRING database to screen for nodes with the highest confidence level (0.900) (**Figure** **2**A). The Autodock vina program was used to perform molecular docking analysis between top 10 proteins and squamocin, revealing that squamocin exhibited the lowest binding energy with the N‐segment peptide chain fragment of 90 kDa heat shock protein (HSP90α, encoded by *HSP90AA1*, Table S1, Supporting Information). HSP90α, an ATPase‐directed chaperone, is one of the most abundant cytosolic molecular chaperones with vital roles in regulating proteostasis.^[^ ^27^ ^]^ The docking results demonstrated that squamocin formed conventional hydrogen bonds with HSP90α at Asn51 (2.45 Å), Gly97 (3.16 Å), Phe138 (3.07 Å), and Tyr139 (1.87 Å) (Figure 2B,C). To validate the computational docking results, differential scanning fluorimetry (DSF) and surface plasmon resonance (SPR) analyses were employed to assess the binding ability of squamocin and HSP90α. Squamocin exhibited a thermal shift (∆T_m_1) from 2.5 to 9.5 °C in the DSF assay, and a K_D_ value of 1.9 × 10^−5^ m in the SPR assay (Figure 2D,E). To verify the predicted binding mode, we generated an HSP90α‐mutant with N51A/G97A/F138K/Y139R and re‐evaluated its binding ability using cellular thermal shift assay (CETSA). The results indicate that this mutant abolished squamocin's binding to HSP90α (Figure 2F), suggesting that HSP90α is a direct target of squamocin. Importantly, we found that knockdown of HSP90α reduced sensitivity to squamocin in HNSCC cell lines (Figure 2G). We also found that the knockdown of HSP90α led to a decrease in both EZH2 and MYC protein levels in SCC15, suggesting that HSP90α is involved in the stabilization of EZH2 and MYC. Furthermore, the degradation of EZH2 and MYC by squamocin was only slightly affected after HSP90α knockdown in SCC15 cells (Figure S6I, Supporting Information), while overexpression of HSP90α impaired the squamocin‐induced degradation of EZH2 and MYC (Figure S6J, Supporting Information). These results suggest that the squamocin‐induced degradation of EZH2 and MYC may largely depend on HSP90α.

Previous studies have demonstrated that in cancer, HSP90α is not only upregulated but also its ATPase activity is increased ≈50‐fold.^[^ ^28^ ^]^ Our pan‐cancer analysis revealed that HSP90α was upregulated across multiple tumor types, and patients with elevated levels of HSP90α experienced poor prognosis, including those with HNSCC (Figure S3, Supporting Information). A recent study found that under cancer cells enduring MYC‐induced stress conditions, HSP90α can form stable epichaperome networks for cancer cell survival, irrespective of tissue of origin or genetic background.^[^ ^17b^ ^]^ These findings suggest that squamocin may exert an inhibitory effect on tumor progression by impairing mitochondrial respiratory Complex I function, reducing ATP production, and disrupting HSP90α function, especially in MYC‐driven tumors.

### 2.3 EZH2 Directly Binds to MYC and Promotes Both the Protein Stability and Transcriptional Activity of MYC in HNSCC

As EZH2 can regulate MYC expression at the transcriptional level or promote MYC stabilization as a non‐PRC2 partner in several tumors,^[^ ^9^, ^13^ ^]^ this prompted us to examine whether squamocin treatment also affected EZH2 levels in two HNSCC cell lines. Notably, squamocin decreased the steady‐state protein level of EZH2 in a dose‐dependent manner without affecting the mRNA level of EZH2 (Figure 2H), suggesting that squamocin regulates EZH2 via a post‐transcriptional mechanism. Following the depletion of EZH2 by squamocin, global H3K27me3 (a mark deposited by EZH2) and MYC levels were also decreased in a dose‐dependent manner (Figure 2H), with both EZH2 and MYC depletion occurring in both cytoplasm and nuclear fraction (Figure 2I). Importantly, overexpression of EZH2 largely rescued the proliferation‐inhibitory effects induced by squamocin, as assessed by CCK8 and colony formation assays (Figure 2J,K). Flow cytometry revealed that EZH2‐overexpression alleviated cell cycle arrest and reduced apoptosis triggered by squamocin (Figure S2C,D, Supporting Information). Collectively, these findings demonstrate that squamocin suppresses tumor growth primarily in an EZH2‐dependent manner in HNSCC.

**Figure 3:**
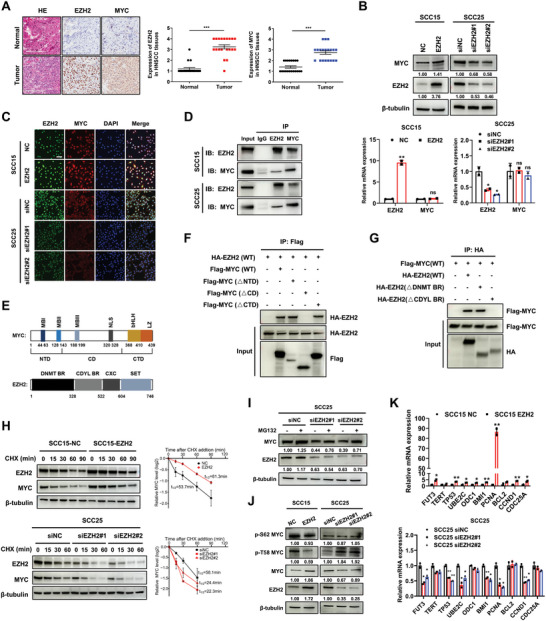
EZH2 directly interacts with MYC in HNSCC. A) IHC staining of HE, EZH2, and MYC in 20 HNSCC patient samples. Representative sections are shown (left panel, scale bars, 200 µm). The intensity of staining of malignant cells was scored to analyze the levels of protein expression (right panel, Wilcoxon matched‐pairs signed rank test). B) The effect of EZH2 on MYC expression was measured by Western blot (upper panel) and qRT‐PCR (lower panel, mean ± SEM; *n* = 2, Student's *t*‐test) in EZH2‐overexpressing SCC15 cells and two siEZH2 (#1 and #2)‐transfected SCC25 cell lines. C) Immunofluorescence staining analysis of the subcellular localization of EZH2 and MYC in the indicated cells. Scale bars: 50 µm. D) Lysates from SCC15 and SCC25 cells were immunoprecipitated with anti‐EZH2 or anti‐MYC and subjected to Western blot. E) Schematic of the domain organization of MYC and EZH2. NTD, CD, and CTD indicate the amino‐terminal, central, and carboxy‐terminal domains, respectively. DNMT BR: DNA methyltransferase‐binding region, CDYL BR: Chromo domain Y‐like protein‐binding region, CXC: Cysteine‐rich domain, SET: Su(var)3–9, enhancer of zeste, trithorax domain. F, G) 293T cells were transfected with plasmids expressing HA‐tagged EZH2, Flag‐tagged MYC, or deletion (Δ) mutants as shown. Anti‐flag‐ (F) or anti‐HA‐ (G) associated precipitates were used to detect EZH2 binding to MYC. H) Effect of EZH2 on the half‐life of MYC protein with 100 µg mL^−1^ CHX treatment as indicated. The graph represents the quantification of MYC protein levels relative to β‐tubulin levels, and the MYC half‐life was calculated using Prism (mean ± SEM; *n* = 2). I) SCC25 cells were transfected with two siEZH2 (#1 and #2) constructs for 48 h, followed by 10 µm MG132 treatment for 8 h. Cell lysates were analyzed by Western blot. J) Western blot analysis of MYC phosphorylation in EZH2‐overexpressing SCC15 cells and siEZH2‐transfected SCC25 cells. K) qRT‐PCR analysis of MYC target genes in EZH2‐overexpressing SCC15 cells (upper panel) and siEZH2‐transfected SCC25 cells (lower panel, mean ± SEM; *n* = 2, Student's *t*‐test). *ns*: non‐significant, ^*^ *p* < 0.05, ^**^ *p* < 0.01, and ^***^ *p* < 0.001.

Next, we investigate whether the oncoproteins EZH2 and MYC cooperate in the progression of HNSCC. Immunohistochemistry (IHC) staining in 20 pairs of HNSCC samples revealed that elevated levels of EZH2 and MYC were present in tumor tissues than adjacent normal tissues (**Figure** **3**A and Table S2, Supporting Information). Notably, depletion of MYC attenuated EZH2‐induced proliferation in EZH2‐overexpressing SCC15 cells, whereas overexpression of MYC exhibited the opposite effect in EZH2‐silenced SCC25 cells (Figure S4, Supporting Information). As the abnormal expression of EZH2 significantly affects MYC protein levels while causing minimal changes in MYC mRNA levels (Figure 3B), we hypothesized that EZH2 promotes MYC accumulation via protein interaction. Immunofluorescence assays revealed the co‐localization of EZH2 and MYC primarily in the nucleus of SCC15 and SCC25 cells, with both upregulation and depletion of EZH2 similarly disrupting the nuclear accumulation of MYC (Figure 3C). Furthermore, reciprocal co‐immunoprecipitation (co‐IP) confirmed the interaction between EZH2 and MYC in two HNSCC cell lines (Figure 3D). We identified that the MYC central domain (CD) and the chromodomain Y‐like protein binding region (CDYL BR) of EZH2 were responsible for their direct interaction (Figure 3E–G). These results establish that MYC is a bona fide interaction partner of EZH2.

We next examined the impact of EZH2 on the protein stability and transcriptional activity of MYC. Notably, time‐course experiments demonstrated that enforced expression of EZH2 extended the half‐life of endogenous MYC protein from 53.7 to 81.3 min, while knockdown of EZH2 accelerated MYC protein turnover, reducing its half‐life from 58.1 to 24.4 and 22.3 min (Figure 3H). Moreover, the loss of MYC induced by EZH2 depletion was efficiently rescued by administration of the proteasome inhibitor MG132 (Figure 3I), indicating that ablation of EZH2 stimulates proteasomal degradation of MYC. A well‐characterized event in MYC degradation involves sequential phosphorylation at two critical residues, serine 62 (p‐S62) and threonine 58 (p‐T58), which result in stabilization and destabilization of MYC, respectively.^[^ ^29^ ^]^ Consistently, ectopically expressed EZH2 had minimal effects on p‐S62 levels but decreased p‐T58 levels, conversely, depletion of EZH2 led to a notable increase in p‐T58 levels (Figure 3J). Additionally, MYC target genes such as *TP53*, *BMI1*, *PCNA*, and *CCND1* were markedly increased in EZH2‐overexpressing SCC15 cells and downregulated in EZH2‐depleted SCC25 cells (Figure 3K), suggesting that EZH2 enhances the transcriptional activity of MYC.

### 2.4 Squamocin Efficiently Suppresses EZH2 Methyltransferase Activity and Promotes MYC Degradation in HNSCC

**Figure 4:**
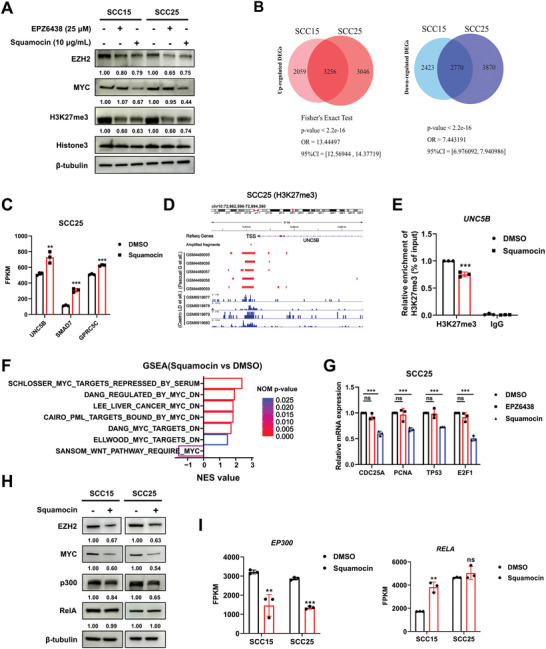
Squamocin efficiently suppresses EZH2 methyltransferase activity and induces MYC degradation in HNSCC. A) Western blot analysis of the corresponding proteins in SCC15 and SCC25 cells following treatment with the indicated drugs for 24 h. B) Venn diagrams showing overlapped up‐ or down‐regulated differentially expressed genes (DEGs, FDR < 0.05) between SCC15 and SCC25 cells after treatment with squamocin. The DEG profiles of the two cell lines were quantified using Fisher's exact test (*p* < 0.05). C) FPKM of indicated genes in RNA‐seq of SCC25 cell lines following treatment with squamocin (mean ± SEM; *n* = 3, Student's *t*‐test). D) The H3K27me3 modification of the *UNC5B* promoter in SCC25 cells was analyzed using the GEO database (GSE149042 and GSE222312). E) ChIP‐qPCR analysis of the enrichment of H3K27me3 on *UNC5B* promoter in SCC25 cells following treatment with 10 µg mL^−1^ squamocin (mean ± SEM; *n* = 3, Student's *t*‐test). F) GSEA analysis of the expression programmers of MYC‐inactivated genes and activated genes in RNA‐seq of SCC15 and SCC25 cells following treatment with squamocin. G) qRT‐PCR analysis of the indicated genes in SCC25 cells treatment with squamocin (10 µg mL^−1^) or EPZ6438 (15 µg mL^−1^) for 24 h (mean ± SEM; *n* = 3, Student's *t*‐test). H) Western blot analysis of the corresponding proteins in SCC15 and SCC25 cells following treatment with the 10 µg mL^−1^ squamocin for 24 h. I) FPKM of indicated genes in RNA‐seq of SCC15 and SCC25 cells following treatment with squamocin (mean ± SEM; *n* = 3, Student's *t*‐test). *ns*: non‐significant, ^*^ *p* < 0.05, ^**^ *p* < 0.01, and ^***^ *p* < 0.001.

As squamocin resulted in reduced levels of H3K27me3 (Figure 2H,I), we hypothesized that it may inhibit the enzymatic function of EZH2. We treated two HNSCC cell lines with 10 µg mL^−1^ squamocin or 25 µm (14 µg mL^−1^) EPZ‐6438, the first enzymatic inhibitor of EZH2 approved by the US Food and Drug Administration (FDA). Notably, although EPZ‐6438 and squamocin exhibited comparable potency in suppressing H3K27me3 levels, only squamocin dramatically inhibited MYC protein accumulation (**Figure** **4**A).

To further investigate the methyltransferase activity of EZH2, we performed a transcriptomic analysis to assess the H3K27me3 landscape in response to squamocin. Comparison of squamocin treatment with DMSO control in SCC15 and SCC25 cells revealed a set of common differentially expressed genes (DEGs) with a false discovery rate (FDR) < 0.05, including 3256 upregulated genes and 2770 downregulated genes (Figure 4B and Table S3, Supporting Information). As anticipated, several canonical EZH2–PRC2 target genes that were transcriptionally suppressed by H3K27me3, such as *UNC5B*, *SMAD7*, and *GPRC5C*, were significantly upregulated in SCC25 cell lines following treatment with squamocin (Figure 4C). Consistently, the ChIP‐qPCR assay confirmed a decrease in global H3K27me3 enrichment on the *UNC5B* promoter due to squamocin treatment in SCC25 cells (Figure 4D,E).

Given the pronounced effect of squamocin on MYC degradation, we investigated whether squamocin regulates the transcriptional activity of MYC. Our RNA‐seq data indicated that the expression programmers of MYC‐inactivated genes were upregulated, whereas MYC‐activated genes were downregulated in SCC15 and SCC25 cells following treatment with squamocin (Figure 4F). qRT‐PCR confirmed the suppression effect of squamocin, but not EPZ‐6438, on MYC‐activated targets (Figure 4G). Collectively, these results demonstrate that squamocin effectively depletes both EZH2 histone methyltransferase and its non‐catalytic binding partner MYC in HNSCC cell lines.

We also investigated whether squamocin could degrade other EZH2's non‐catalytic binding partners, such as p300^[^ ^30^ ^]^ and RelA.^[^ ^31^ ^]^ The results indicated that the accumulation of p300, but not RelA (p65), was reduced in two HNSCC cells treated with squamocin (Figure 4H). Furthermore, we observed that the mRNA levels of *EP300* (encoding p300) were significantly downregulated by squamocin, while *RELA* (encoding RelA) was either significantly upregulated or remained unaffected in both HNSCC cell lines (Figure 4I). These results demonstrated that squamocin has minimal impact on the degradation of other non‐catalytic binding partners of EZH2, including p300 and RelA.

### 2.5 Squamocin Effectively Degrades EZH2 and its Non‐PRC2 Partner MYC by Facilitating Endoplasmic Reticulum (ER) Stress‐Associated Protein Ubiquitylation and Degradation

**Figure 5:**
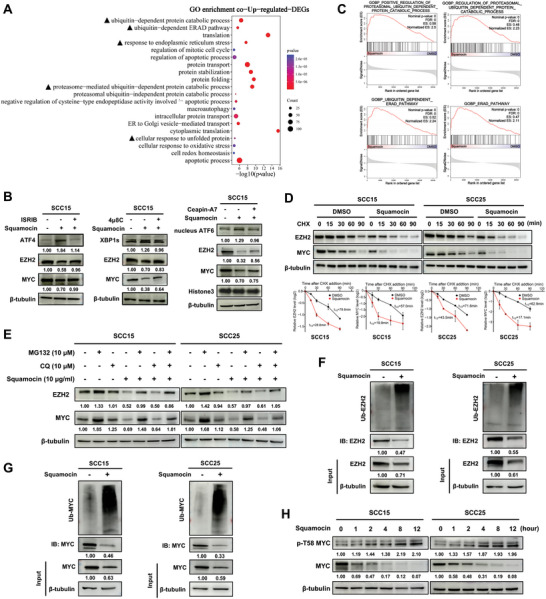
Squamocin activates ER stress‐associated protein ubiquitination and degradation. A) Scattergrams of gene ontology enrichment analysis for common upregulated DEGs between SCC15 and SCC25 cells after treatment with squamocin. GO with the top 20 biological processes were shown (*p* < 0.05). B) Western blot analysis of EZH2 and MYC in SCC15 cells treated with 10 µg mL^−1^ squamocin for 24 h and with ER stress inhibitors (ISRIB: 20 µm, 4µ8C: 20 µm, CeapinA7: 20 µm) for 12 h. C) GSEA showing the regulation of proteasomal ubiquitin‐dependent protein catabolic process and ERAD pathway in SCC15 and SCC25 cells with squamocin treatment. D) SCC15 and SCC25 cells were first treated with DMSO or 10 µg mL^−1^ squamocin for 24 h, before subjecting them to 100 µg mL^−1^ CHX treatment for indicated time. Western blot was performed (upper panel) and the degradation curves of EZH2 and MYC were plotted (lower panel). The half‐lives of EZH2 and MYC were calculated using Prism (mean ± SEM; *n* = 2). E) SCC15 and SCC25 cells were treated with 10 µg mL^−1^ squamocin for 24 h, 10 µm MG132 for 8 h, 10 µm CQ for 8 h, or squamocin for 24 h followed by MG132 or/and CQ treatment for 8 h. Cell lysates were analyzed by Western blot. F, G) Western blot analysis of the ubiquitination of EZH2 (F) or MYC (G) in SCC15 and SCC25 cells treated with 10 µg mL^−1^ squamocin vs DMSO for 24 h. H) SCC15 and SCC25 cells were treated with 10 µg mL^−1^ squamocin for the indicated time periods, followed by immunoblot analysis.

MYC‐hyperactivated tumor cells exhibit enhanced activation of the UPR in multiple human cancers.^[^ ^16^ ^]^ Consistent with this notion, persistent, non‐lethal UPR signaling has been demonstrated in both in vitro and in vivo models of HNSCC,^[^ ^32^ ^]^ representing a potential therapeutic target that cancer cells with a constitutively active UPR are highly susceptible to additional ER stress, which can trigger apoptosis.^[^ ^16^ ^]^ Importantly, we observed that squamocin not only inhibited the mitochondrial respiratory Complex I, leading to increased reactive oxygen species (ROS) and reduced ATP levels but also impaired the binding of HSP90α and ATP, both of which synergistically promote ER stress and the UPR. As expected, gene ontology (GO) analysis revealed that common upregulated DEGs were significantly enriched in “response to ER stress” and “response to unfolded protein (UPR)” (**Figure** **5**A and Table S4, Supporting Information). Gene set enrichment analysis (GSEA) confirmed that squamocin treatment resulted in significant upregulation of ER stress and the UPR (Figure S5A, Supporting Information). In mammalian cells, the UPR is initiated by three ER transmembrane proteins that operate as sensors of ER stress: activating transcription factor 6 (ATF6), inositol‐requiring enzyme 1α (IRE1α), and PRKR‐like ER kinase (PERK).^[^ ^33^ ^]^ Consistently, we found that squamocin treatment enhanced the accumulation of three ER stress sensors XBP1s, ATF4, and ATF6 in SCC15 and SCC25 cell lines, blocking any of these sensors with specific inhibitors markedly blunted the degradation of EZH2 and MYC induced by squamocin (Figure 5B; Figure S5B, Supporting Information). Additionally, common upregulated DEGs were significantly enriched for the “apoptotic process” (Figure 5A and Table S4, Supporting Information), supporting the apoptosis phenotype induced by squamocin (Figure 1D,E).

The ubiquitin‐proteasome system (UPS) and autophagy have been reported to regulate the degradation of misfolded proteins, thereby maintaining ER homeostasis.^[^ ^33^ ^]^ Consistently, our RNA‐seq data indicated that common upregulated DEGs were significantly enriched in “ubiquitin‐dependent protein catabolic processes” and “ubiquitin‐dependent ERAD pathway” (Figure 5A and Table S4, Supporting Information), as verified by GSEA (Figure 5C). To determine which pathway is involved in squamocin‐induced EZH2 and MYC degradation, we first conducted a time‐course analysis confirming that squamocin significantly shortened the half‐lives of endogenous EZH2 and MYC proteins (Figure 5D). Second, the decrease in EZH2 and MYC protein levels was efficiently rescued by the proteasome inhibitor MG132; however, administration of the autophagy inhibitor chloroquine (CQ) did not yield similar results (Figure 5E; Figure S5C,D, Supporting Information), suggesting that squamocin‐mediated degradation of EZH2 and MYC occurs via UPS pathways. Third, the ubiquitination assay validated that both EZH2 and MYC proteins were ubiquitinated and degraded by squamocin in two HNSCC cell lines (Figure 5F,G). Similar to the deletion of EZH2 by siRNA (Figure 3J), we also observed a notable increase in MYC p‐T58 following squamocin treatment in both SCC15 and SCC25 cells (Figure 5H). Cytotoxic drugs can induce ER stress and activate the ERAD, however, how ERAD machinery degrades diverse substrates remains a long‐standing question.^[^ ^34^ ^]^ Finally, we investigated whether other compounds known to induce ER stress would similarly affect both EZH2 and MYC degradation as seen with squamocin. Interestingly, our results demonstrated that tunicamycin (TM), a well‐established ER stress inducer,^[^ ^35^ ^]^ failed to promote ERAD for both EZH2 and MYC (Figure S5E, Supporting Information), implying that substrate specificity for ERAD may be dependent on stress conditions as well as tumor type. Taken together, these findings indicate that squamocin activates ER stress and promotes ubiquitination of both EZH2 and MYC for subsequent proteasomal degradation.

### 2.6 Squamocin Mediates the Degradation of Both EZH2 and MYC Through the UBA6‐UBE2Z‐FBXW7 Ubiquitin Cascade in HNSCC

**Figure 6:**
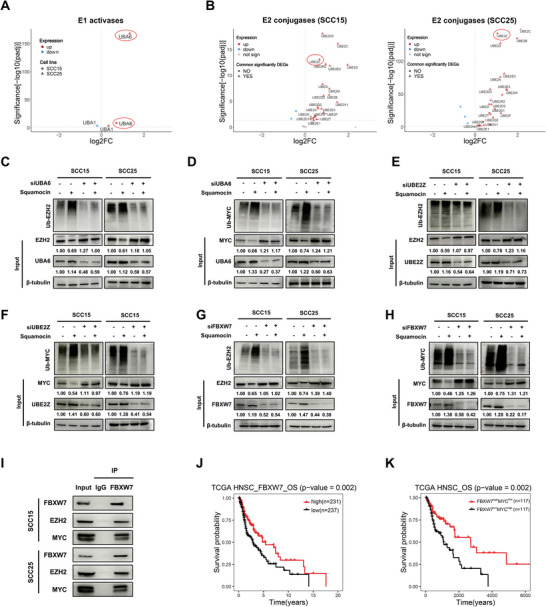
Squamocin enhances UBA6‐UBE2Z‐FBXW7 ubiquitin cascade‐mediated degradation of EZH2 and MYC. A, B) Volcano plots showing DEGs (FDR < 0.05) encoding E1s (A) and E2s (B) based on RNA‐seq profiles of SCC15 and SCC25 cells with squamocin treatment relative to the DMSO control. C–H) SCC15 and SCC25 cells were transfected with siUBA6 (C and D), siUBE2Z (E and F), or siFBXW7 (G and H) for 48 h followed by squamocin (10 µg mL^−1^) treatment for 24 h. Cell lysates were immunoprecipitated with anti‐EZH2 or anti‐MYC and analyzed with anti‐ubiquitin. I) Co‐immunoprecipitation for endogenous FBXW7 interaction with EZH2 or MYC in SCC15 and SCC25 cells. J) Overall survival of patients with TCGA‐HNSC stratified by high or low expression of FBXW7 (optimal cutoff level was determined using the R survminer package) was estimated by the Kaplan–Meier method and compared using the Log‐rank test. K) Overall survival of patients with TCGA‐HNSC stratified by high or low expression of the indicated genes (median values as cutoff) was estimated by the Kaplan–Meier method and compared using the Log‐rank test.

Classically, ubiquitylation is achieved by a sequential enzymatic cascade of E1 activases (E1s), E2 conjugates (E2s), E3 ligases (E3s), and deubiquitinases (DUBs).^[^ ^36^ ^]^ To decipher the specific molecular mechanism underlying squamocin‐mediated degradation of both EZH2 and MYC, we analyzed the global UPS in our RNA‐seq data. Notably, many human ubiquitin enzyme genes were upregulated by squamocin (FDR < 0.05, **Figure** **6**A,B and Table S5, Supporting Information). We validated the RNA‐seq results by conducting qRT‐PCR analyses on two HNSCC cell lines, which demonstrated that squamocin significantly upregulated a set of key UPS genes including E1s (UBA6), E2s (UBE2Z, UBE2B, UBE2E2, UBE2E3 and UBE2S), and E3s (FBXW7) (Figure S6A, Supporting Information). Notably, these gene expressions were reduced following treatment with ER stress inhibitors (Figure S6A, Supporting Information), supporting the notion that squamocin reprograms the UPS by triggering ER stress.

Currently, only two human E1s, known as UBA1 and UBA6, have been identified as initiators of the UPS. Notably, we found that UBA6 but not UBA1 was co‐upregulated in both HNSCC cell lines (Figure 6A). Knockdown of UBA6 abolished the squamocin‐mediated ubiquitination and degradation of both EZH2 and MYC (Figure 6C,D), while silencing UBA1 had minimal impact (Figure S6B, Supporting Information), suggesting that squamocin activates the UPS via UBA6 rather than UBA1 in HNSCC. UBA6 is a dual‐activity E1s enzyme that activates both ubiquitin and the ubiquitin‐like protein FAT10, which targets proteins for rapid proteasomal degradation.^[^ ^37^ ^]^ However, we observed no significant effect of squamocin treatment on FAT10 modification in these two HNSCC cell lines (Figure S6C, Supporting Information). As UBE2Z (UBA6‐specific E2s) was significantly co‐upregulated in both squamocin‐treated HNSCC cell lines (Figure 6B), we next investigated whether UBE2Z could mediate the degradation of both EZH2 and MYC. The results indicated that squamocin failed to decrease levels of EZH2 and MYC in UBE2Z knockdown HNSCC cell lines (Figure 6E,F), suggesting that enhancement of the UBA6‐UBE2Z cascade is responsible for squamocin‐induced degradation of both EZH2 and MYC protein.

Specific E3s and DUBs provide recognition specificity to the ubiquitin pathway via direct interaction with the substrates. We matched our gene expression profile with validated or predicted E3s and DUBs targeting EZH2 and MYC using the UbiBrowser_v2 database.^[^ ^38^ ^]^ Notably, following squamocin treatment, 23 genes encoding E3s and 17 genes encoding DUBs were found to be dysregulated in both HNSCC cell lines (Figure S6D, Table S6, Supporting Information). Cluster analysis of the heatmap revealed that DUBs (USP28, USP37, USP33 and USP22), BTRC and SKP2, which are known to stabilize EZH2 or MYC, were downregulated, while E3s responsible for degrading EZH2 or MYC, including NEDD4L, PML, FBXO8, FBXW7 and RING1, were increased by squamocin treatment (Figure S6E, Table S7, Supporting Information). qRT‐PCR demonstrated that FBXW7 and RING1 were the most significantly upregulated E3s after squamocin treatment (Figure S6F, Supporting Information). Using specific siRNAs, we observed that FBXW7 knockdown distinctly blocked squamocin‐induced degradation of both EZH2 and MYC, while RING1 knockdown had minimal impact (Figure 6G,H; Figure S6G,H, Supporting Information), suggesting that squamocin primarily mediates degradation of both EZH2 and MYC in an FBXW7‐dependent manner. Co‐IP assay confirmed the binding of FBXW7 to both EZH2 and MYC in these two HNSCC cell lines (Figure 6I). Additionally, survival analysis indicated that a high level of FBXW7 was associated with a promising prognosis in HNSCC patients (Figure 6J), and patients with high FBXW7 expression coupled with low MYC expression had a better prognosis than those with low FBXW7 expression alongside high MYC expression (Figure 6K). Furthermore, we found that squamocin treatment led to an increase in the expression levels of UBA6, UBE2Z, and FBXW7 in SCC15 cells. Additionally, the knockdown of HSP90α blunted squamocin‐induced upregulation of the UBA6‐UBE2Z‐FBXW7 pathway, while HSP90α overexpressing attenuated the activation of squamocin on this pathway (Figure S6I,J, Supporting Information). Together, these results suggest that HSP90α is involved in the regulation of the UBA6‐UBE2Z‐FBXW7 ubiquitin cascade by squamocin.

Collectively, these findings indicate that squamocin activates the UPS via UBA6 (E1), facilitating ubiquitin charging to UBE2Z (E2) and increasing the activities of FBXW7 (E3) to degrade both EZH2 and MYC, in an HSP90α‐dependent manner in HNSCC.

### 2.7 Squamocin Suppresses the EZH2/MYC Axis in Multiple Tumors

As EZH2 and MYC were dysregulated in a range of human tumors,^[^ ^11^, ^12^ ^]^ we sought to determine whether squamocin can effectively degrade both EZH2 and MYC in other types of cancer. Our analysis using the TCGA database revealed that EZH2 and MYC were upregulated across multiple human tumors, including stomach adenocarcinoma (STAD), colon adenocarcinoma (COAD), and rectum adenocarcinoma (READ) (Figure S7A, Supporting Information). Importantly, GSEA analysis indicated that tumors with high levels of EZH2 were positively correlated with the expression programmers of MYC‐activated genes, but negatively correlated with MYC‐inactivated genes (Figure S7B, Supporting Information). These results underscore the universality of the EZH2/MYC axis in multiple cancer types. Activation of MYC in these tumors may render them sensitive to squamocin treatment. Importantly, cancers with MYC activation are highly sensitive to HSP90 inhibitors such as PU‐H71, an ATP competitor similar in function to squamocin.^[^ ^17b^ ^]^ As anticipated, we observed dose‐ and time‐dependent inhibition of cell viability by squamocin treatment in GC (AGS and MNK45) and CRC (SW480 and LOVO) cell lines, with IC_50_ was calculated in AGS (IC_50_ = 13.60 µg mL^−1^) and MNK45 (IC_50_ = 12.43 µg mL^−1^), SW480 (IC_50_ = 12.04 µg mL^−1^) and LOVO (IC_50_ = 13.72 µg mL^−1^), respectively (Figure S7C,D, Supporting Information). Furthermore, global H3K27me3 and MYC levels were decreased by squamocin treatment in GC and CRC cell lines (Figure S7E, Supporting Information). Ubiquitination assay confirmed that both EZH2 and MYC underwent ubiquitination followed by degradation mediated by squamocin treatment in GC and CRC cell lines (Figure S7F,G, Supporting Information).

To elucidate the mechanism by which squamocin degrades both EZH2 and MYC in CRC and GC cells, we assessed the impact of inhibiting ER stress response after squamocin treatment. Notably, blocking ER stress response using specific inhibitors significantly attenuated the degradation effects on both EZH2 and MYC induced by squamocin (Figure S7H–J, Supporting Information), suggesting that squamocin also enhances ER‐associated protein degradation processes within GC and CRC contexts. We subsequently investigated the role of the UBA6‐UBE2Z‐FBXW7 ubiquitin cascade in mediating this degradation process induced by squamocin within GC and CRC cells. Similarly to our observations from HNSCC cell lines, knockdown of any of the UBA6‐UBE2Z‐FBXW7 ubiquitin cascade with specific siRNA blunted the degradation of both EZH2 and MYC in GC and CRC cell lines (Figure S7K–P, Supporting Information).

### 2.8 Squamocin Effectively Inhibits Tumor Growth of Multiple In Vivo Tumor Models

**Figure 7:**
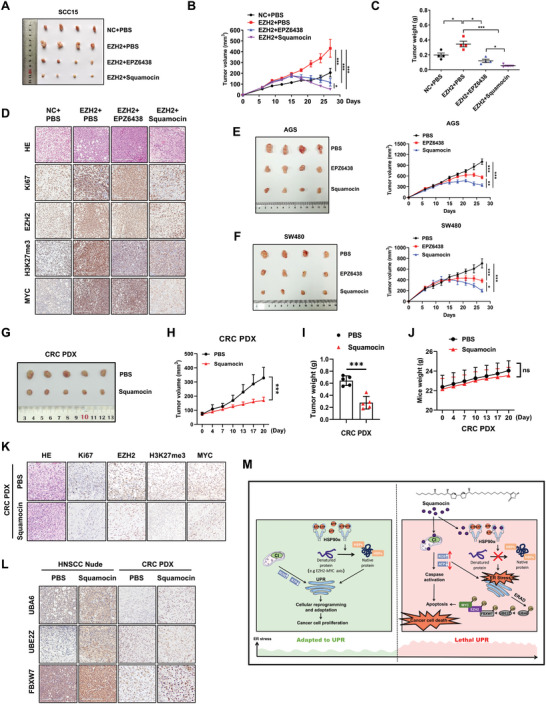
Squamocin effectively suppresses multiple tumor growth in vivo. A) Representative tumor images (*n* = 4) of HNSCC xenografts in nude mice. B) Plots of tumor volumes measured every 3 days (mean ± SEM; *n* = 4, two‐way ANOVA test). C) Summary of tumor weight harvested after euthanasia (mean ± SEM; *n* = 4, Student's *t*‐test). D) IHC staining of HE, Ki67, EZH2, H3K27me3, and MYC in the tumor xenografts. Scale bars: 200 µm. E, F) Representative tumor images and tumor volumes of GC (E) and CRC (F) xenografts in nude mice (mean ± SEM; *n* = 4, two‐way ANOVA test). G) Representative tumor images of CRC PDX xenografts in NGS mice. H, I) Plots of tumor volumes (H) measured every 3 days (mean ± SEM; *n* = 5, two‐way ANOVA test), and summary of tumor weight (I) harvested after euthanasia (mean ± SEM; *n* = 5, Student's *t*‐test). J) Weight changes of CRC PDX mice throughout the experiment (mean ± SEM; *n* = 5, two‐way ANOVA test). K) IHC staining of HE, Ki67, EZH2, H3K27me3, and MYC in CRC PDX xenografts. Scale bars: 50 µm. L) IHC staining of UBA6, UBE2Z, and FBXW7 in HNSCC and CRC tumor xenografts. Scale bars: 200 µm. M) Model depicting the regulatory mechanism of EZH2 and MYC by squamocin through UBA6‐UBE2Z‐FBXW7 ubiquitin cascade. ^*^ *p* < 0.05, ^**^ *p* < 0.01, and ^***^ *p* < 0.001.

To investigate whether squamocin could inhibit tumor growth by targeting the EZH2‐MYC axis in vivo, we generated a nude mouse xenograft model of HNSCC with stable EZH2 overexpression. Mice bearing EZH2‐overexpression or control SCC15 tumors were treated intraperitoneally with phosphate‐buffered saline (PBS), EPZ‐6438, or squamocin every 3 days for a total of five times. Consistent with the in vitro findings, overexpression of EZH2 resulted in a significant promotion in tumor volume and weight, which were effectively abrogated by treatment with EPZ‐6438 or squamocin (**Figure** **7**A–C). The tumor growth inhibition rates (TIR) of squamocin and EPZ‐6348 were 84.06% and 65.22%, respectively (**Table** **1**). IHC staining of xenografts demonstrated that EZH2 overexpression was accompanied by increased Ki67, H3K27me3, and MYC levels. Similar to the EPZ‐6438 treatment, squamocin distinctly decreased levels of EZH2, Ki67, and H3K27me3, but only tumors treated with squamocin exhibited pronounced loss of MYC (Figure 7D). Collectively, these results underscore the potential advantage of squamocin over inhibitors targeting the enzymatic function of EZH2 in deleting MYC, suggesting that squamocin not only inhibits the catalytic activity of EZH2 but also suppresses its noncanonical activities.

**Table 1:** In vivo antitumor effects of different drug groups (HNSCC).

Group	Tumor weight [g]	Inhibition rate [%]
EZH2+PBS	0.3450 ± 0.07550	NA
EZH2+EPZ‐6438 (50 mg kg^−1^)	0.1200 ± 0.04243^*^	65.22
EZH2+Squamocin (0.4 mg kg^−1^)	0.0550 ± 0.01732^***#^	84.06

Tumor weight results are presented as mean ± SEM, n = 4.

^*^ *p* < 0.05, ^***^ *p* < 0.001 vs EZH2+PBS.

# *p* < 0.05 vs EZH2+EPZ‐6438.

Subsequently, we evaluated the effects of squamocin in additional in vivo tumor models. The antitumor efficacy of squamocin was assessed using AGS and SW480 xenograft models, demonstrating that squamocin effectively inhibited tumor growth in both GC with a TIR of 56.49%, and CRC with a TIR of 53.82%. In contrast, the TIR of EPZ‐6438 in GC and CRC were 32.7% and 27.43%, respectively (Figure 7E,F and **Tables** **2** and **3**). Furthermore, squamocin demonstrated a favorable safety profile in mouse models, as no significant histological changes were observed in vital organs including the heart, liver, spleen, lungs, and kidneys (Figure S8A, Supporting Information). Additionally, subcutaneous patient‐derived xenograft^[^ ^39^ ^]^ mouse models of CRC were established to determine the in vivo anticancer activities of squamocin. As expected, we found that tumor volumes and weight in the squamocin treatment group were significantly reduced compared to those in the control group (Figure 7G–I), with a TIR of 56.83% (**Table** **4**), and no associated loss of body weight due to treatment (Figure 7J). Decreased levels of Ki67, EZH2, H3K27me3, and MYC were detected in squamocin treatment group (Figure 7K). Importantly, IHC staining of xenografts demonstrated that squamocin distinctly increased levels of UBA6‐UBE2Z‐FBXW7 cascade in both nude xenografts of HNSCC and PDX xenografts of CRC (Figure 7L), supporting the in vitro findings. PDX models accurately recapitulate human tumors with high fidelity and exhibit treatment responses consistent with those observed clinically.^[^ ^39^ ^]^ To validate this, we subjected our PDX and the primary human CRC tumor samples to a series of genomic comparisons. Whole exome sequencing (WES) analysis revealed that PDX largely recapitulated the primary tumor from which they were derived in terms of copy number variations (CNVs) (Figure S9, Supporting Information). Collectively, these results indicate that, at least in HNSCC and gastrointestinal cancer, squamocin demonstrates potential therapeutic efficacy against EZH2/MYC axis‐driven tumors without causing significant host toxicity.

**Table 2:** In vivo antitumor effects of different drug groups (GC).

Group	Tumor weight [g]	Inhibition rate [%]
PBS	0.9250 ± 0.04173	NA
EPZ‐6438 (50 mg kg^−1^)	0.6225 ± 0.04460^**^	32.70
Squamocin (0.4 mg kg^−1^)	0.4025 ± 0.03637^***##^	56.49

Tumor weight results are presented as mean ± SEM, n = 4.

^**^ *p* < 0.01, ^***^ *p* < 0.001 vs PBS.

## *p* < 0.01 vs EPZ‐6438.

**Table 3:** In vivo antitumor effects of different drug groups (CRC).

Group	Tumor weight [g]	Inhibition rate [%]
PBS	0.7200 ± 0.04143	NA
EPZ‐6438 (50 mg kg^−1^)	0.5225 ± 0.02720^**^	27.43
Squamocin (0.4 mg kg^−1^)	0.3325 ± 0.02056^***##^	53.82

Tumor weight results are presented as mean ± SEM, n = 4.

^**^ *p* < 0.01, ^***^ *p* < 0.001 vs PBS.

## *p* < 0.05 vs EPZ‐6438.

**Table 4:** In vivo antitumor effect of squamocin in CRC PDX models.

Group	Tumor weight [g]	Inhibition rate [%]
PBS	0.6440 ± 0.03572	NA
Squamocin (0.4 mg kg^−1^)	0.2780 ± 0.04705^***^	56.83

Tumor weight results are presented as mean ± SEM, n = 5.

^***^ *p* < 0.001.

A previous study showed the neurotoxicity of annonacin, a compound of the ACGs family.^[^ ^40^ ^]^ To assess the neurotoxicity of squamocin in vivo, toxicology experiments were performed using Wister rats. Rotenone,^[^ ^41^ ^]^ a well‐known mitochondrial respiratory Complex I inhibitor that can induce potent neurotoxicity was used as a positive control. Rats received intravenously injected squamocin at a high dose of 80 µg kg^−1^, a middle dose of 32 µg kg^−1^ or a low dose of 12.8 µg kg^−1^, and rotenone at a high dose of 20 µg kg^−1^ every day for one week, respectively. The results indicated that the structure of the brain, morphology, and Nissl bodies of neurons were normal in the control group; however, there was a significant reduction in the number of Nissl bodies within the rotenone treatment group. In contrast, squamocin treatment caused minimal neuronal damage, with no significant differences observed across varying concentrations of squamocin (Figure S8B–D, Supporting Information). Additionally, both squamocin and rotenone did not significantly affect liver and kidney morphology or function as assessed by hematoxylin‐eosin (HE) staining and blood biochemical analyses (Figure S8E,F, Supporting Information). Overall, these findings suggest that squamocin is less neurotoxic than rotenone and represents an attractive candidate for targeting EZH2/MYC axis‐dependent cancers.

## 3 Discussion

In this study, we have taken advantage of the expanding antitumor effects of squamocin in tumors driven by the EZH2/MYC axis and elucidated the underlying mechanisms behind these effects. By identifying HSP90α as a direct target of squamocin, we reveal a novel mechanism through which squamocin not only inhibits mitochondrial respiratory Complex I, resulting in reduced ATP production and increased ROS accumulation but also disrupts the binding of HSP90α to ATP. These tumor cell‐intrinsic events caused by squamocin provoked ER stress and the UPR, leading to ERAD‐mediated degradation of EZH2 and MYC, as well as apoptosis. Importantly, our observations uncover a novel ubiquitination cascade composed of UBA6, UBE2Z, and FBXW7 that may regulate the degradation of the squamocin‐induced EZH2/MYC axis in pan‐gastrointestinal tumors (Figure 7M).

EZH2 has emerged as a critical regulator by forming a complex with MYC and promotes MYC protein stability through its enzymatic or non‐enzymatic activity in various tumors.^[^ ^13^, ^30^ ^]^ However, the specific mechanism by which EZH2 influences MYC stability appears to be tumor‐type dependent. Therefore, the interaction patterns between EZH2 and MYC in human tumors require meticulous investigation. In HNSCC, a high level of EZH2 or MYC has been associated with tumorigenesis, recurrence, lymph node metastasis, and poor prognosis.^[^ ^42^ ^]^ However, the precise cooperative roles of these oncoproteins in HNSCC progression remain unclear. Although MYC can act as a PRC2‑regulated target gene,^[^ ^9^ ^]^ we did not observe EZH2 regulating MYC at the transcriptional level. By contrast, we identified EZH2 as a direct, high‐confidence MYC binding partner, which was crucial for malignant growth in HNSCC. We showed that EZH2 directly interacts with MYC via the CD in two HNSCC cell lines, but not MB1 (MYC homology box 1 domain) and MB2. Notably, the ectopically expressed EZH2 decreased MYC p‐T58 levels, whereas depletion of EZH2 by siRNA or treatment with squamocin resulted in a significant increase in MYC p‐T58 levels. MYC p‐T58 is recognized by E3 ubiquitin ligases and degraded by the 26S proteasome.^[^ ^43^ ^]^ Consistently, we found that E3 ligase FBXW7 binds to the endogenous EZH2 and MYC in the two HNSCC cell lines. Our findings indicate that EZH2 enhances MYC protein stability by modulating its p‐T58. However, Wang et al.^[^ ^13c^ ^]^ demonstrated that EZH2 directly interacted with MYC in neuroblastoma via MB2, and that ectopically expressed EZH2 has minimal effects on the MYC p‐T58 levels, suggesting that stabilization of MYC by EZH2 occurs via a MYC p‐T58‐independent mechanism. Thus, the interaction patterns between EZH2 and MYC, as well as the mechanisms by which EZH2 promotes the stabilization of the EZH‐MYC complex, vary across tumor types and warrant thorough investigation.

Our study demonstrated that squamocin effectively suppresses the functions of the EZH2/MYC axis across multiple cancer types. Current EZH2 inhibitors, such as EPZ‐6438, which only inhibit the catalytic activity of EZH2, may be insufficient to attenuate its noncanonical activities. In contrast, squamocin demonstrates a more pronounced suppression of tumor growth both in vitro and in vivo, exerting significant inhibition on H3K27me3 and MYC, which represent the enzymatic and non‐catalytic functions of EZH2, respectively. Interestingly, MS177, a recently developed EZH2‐specific PROTAC degrader by other groups,^[^ ^13b^ ^]^ achieved effective depletion of both EZH2 and interacting partners MYC in leukemia. In contrast to squamocin‐mediated MYC degradation, which depends on the E3 ligase FBXW7, MS177‐mediated MYC degradation is independent of all tested E3 ligases, including FBXW7, UBR5, SKP2, and HUWE1, potentially leading to off‐target effects.

As MYC‐hyperactivated activated cells exhibit enhanced activation of the UPR in various human cancers, which can facilitate tumor survival,^[^ ^16^ ^]^ the mechanisms underlying this effect remain largely unknown. Interestingly, tumor‐derived HSP90 is found exclusively within multichaperone complexes exhibiting high ATPase activity, whereas HSP90 from normal tissues remains in a latent, uncomplexed state,^[^ ^28^ ^]^ however, the underlying mechanism remains unclear. A recent study demonstrated that under tumor cells enduring MYC‐induced stress conditions, HSP90, including HSP90 α/β, can form a network of stable, survival‐facilitating high‐molecular‐weight complexes to enhance tumor survival, regardless of tissue of origin or genetic background.^[^ ^17b^ ^]^ Importantly, HSP90 inhibitors such as PU‐H71 and 17‐AAG, which function as ATP competitors like squamocin, exhibit a higher binding affinity for HSP90 derived from tumor cells compared to that from normal cells; furthermore, tumors with MYC activation are particularly sensitive to these HSP90 inhibitors.^[^ ^17^, ^28^ ^]^ Consistent with this notion, we found that the EZH2/MYC axis is universally active across multiple cancer types, and squamocin promotes significant ER stress in these tumors; consequently, it promotes ERAD of EZH2 and its partner MYC via the UBA6‐UBE2Z‐FBXW7 ubiquitin cascade, ultimately resulting in tumor growth arrest. These findings broaden our understanding of HSP90 as a mechanism that not only improves the survival of tumors with MYC activation but also likely explains the strong antitumor effect that our observation in multiple tumor models.

Cytotoxic drugs can induce ER stress and activate the ERAD;^[^ ^16^ ^]^ however, how ERAD machinery recognizes substrates through specific ERAD E3 ligases remains a long‐standing question. A previous study demonstrated that three well‐established ER stress inducers, including brefeldin A (BFA), tunicamycin (TM), and thapsigargin (TG), promote the ubiquitination and degradation of SLC1A5/38A2 in breast cancer cells.^[^ ^44^ ^]^ However, it has been reported that TM induces the expression of SLC1A5/38A2 in pancreatic β‐cells.^[^ ^45^ ^]^ Another study by Wang and colleagues^[^ ^46^ ^]^ demonstrated that secoemestrin C induces ER stress and promotes ERAD‐mediated YAP degradation in pancreatic adenocarcinoma. Interestingly, our results indicate that squamocin, rather than TM, induces ERAD of both EZH2 and MYC. Thus, these findings suggest that the specificity of ERAD for certain substrates may depend on stress conditions and tumor type.

In summary, our data elucidate the role of squamocin in the degradation of both EZH2 and MYC across multiple tumor models. Given the universal involvement of the EZH2/MYC axis in tumorigenesis, this class of inhibitors has the potential to accelerate the development of therapeutics targeting cancers driven by the EZH2/ MYC axis.

## 4 Experimental Section

### Cell Culture and Clinical Samples

The human HNSCC cell lines SCC15 and SCC25, gastric cancer (GC) cell lines AGS and MNK45, colorectal cancer (CRC) cell lines SW480 and LOVO, and the human embryonic kidney cell line HEK293T cells were obtained from the American Type Culture Collection (ATCC, USA). All cell lines were maintained in F12/DMEM medium (Gibco, USA) supplementary with 10% fetal bovine serum (Gibco, USA), 10 mm HEPES, 1 mm sodium pyruvate, 100 units mL^−1^ penicillin, and 100 units mL^−1^ streptomycin (Gibco, USA). They were grown in a humidified atmosphere consisting of 5% CO_2_ and 95% air at 37 °C. The human HNSCC tissues (*n* = 20) were obtained at the Department of Stomatology of Nanfang Hospital, Southern Medical University. Approvals from the ethical committee of the Institutional Review Board for Human Use at Nanfang Hospital and prior patient's consent were previously obtained for the use of these clinical specimens for research purposes.

A detailed description of all methods used in this study can be found in the Supporting Information.

### Data Availability Statement

All data needed to evaluate the conclusions in the paper are present in the paper and/or the Supplementary Materials. In addition, all RNA‐Seq raw and processed datasets were submitted to the GEO under the accession number GSE245065.

## Conflict of Interest

The authors declare no conflict of interest.

## Author Contributions

Y.Z., Y.L., X.W., and Z.C. contributed equally to this work. Y.S.N. and Y.Z performed the conceptualization. Y.Z., Y.R.L., X.T.W., and Z.F.C. did methodology. Y.Z., Y.R.L., X.T.W., Z.F.C., B.J.C., B.X.H., T.E.T, H.R.C., and X.L.L. did investigation. Y.Z. and Y.R.L performed statistical analysis. Y.Z. wrote the original draft. Y.S.N. did review and editing. X.T.W. and Z.F.C. arranged resources. Y.S.N. did supervision. Y.S.N. performed funding acquisition All authors contributed to the data acquisition and interpretation. All authors reviewed and approved the manuscript.

## Supporting information



Supporting Information

Supplemental Table 1

Supplemental Table 3

Supplemental Table 4

Supplemental Table 5

Supplemental Table 6

Supplemental Table 7

## Data Availability

The data that support the findings of this study are available in the supplementary material of this article.
