# Supplementary material for: Squamocin Suppresses Tumor Growth through Triggering an Endoplasmic Reticulum Stress‐Associated Degradation of EZH2/MYC Axis
Source: Adv Sci (Weinh). 2025 Jan 17;12(15):2413120. doi: 10.1002/advs.202413120 (PMC12005766; doi:10.1002/advs.202413120)
Supplement: Supplementary file 1 — Supporting Information [file ADVS-12-2413120-s006.pdf]

ADVANCED SCIENCE
Open Access

Supporting Information

for *Adv. Sci.*, DOI 10.1002/advs.202413120

Squamocin Suppresses Tumor Growth through Triggering an Endoplasmic Reticulum Stress-Associated Degradation of EZH2/MYC Axis

*Yin Zhu, Yurui Liu, Xiangtao Wang, Zhifeng Chen, Baojian Chen, Bingxin Hu, Tiane Tang, Haoran Cheng, Xinglong Liu and Yunshan Ning**

The file includes:

1. **Supplementary Materials and Methods**
2. **Figures S1 to 9**
3. **Tables S2, S8 to 10**
4. **Supplementary References**

1. Supplementary Materials and Methods

Chemicals

Squamocin was isolated from the seed of Annona squamosa according to the procedure reported [1], and the structure of the obtained squamocin was elucidated using ESI-MS and NMR. Squamocin (20 mg/mL) was dissolved in DMSO to make stock solutions. These stock solutions were kept at −80°C and thawed immediately before each experiment. EPZ-6438 (100 mM, Selleck, #S7128), cycloheximide (CHX, 100 mg/mL, MedChemExpress, #HY-12320), MG132 (10 mM, MedChemExpress, #HY-13259), chloroquine (CQ, 10 mM, TargetMol, #T8689), ISRIB (10 mM, MedChemExpress, #HY-12495A), 4μ8C (10 mM, MedChemExpress, #HY-19707), ceapinA7 (10 mM, MedChemExpress, #HY-108434), rotenone (50 mg/mL, MedChemExpress, #HY-B1756) and mito-TEMPO (10 mM, MedChemExpress, #HY-112879) were dissolved in DMSO as stock solutions, respectively. The stock solutions were kept at −80°C and thawed immediately before each experiment.

Lentivirus transduction and RNA interference

The EZH2 and MYC plasmids were generated by subcloning PCR-amplified full-length human EZH2 (NM_001203247.2) and MYC (NM_001354870.1) cDNA into the pOZ-FH-C-puro plasmid (Addgene, #32516) or pRRLSIN.cPPT.PGK-GFP.WPRE (Addgene, #12252), respectively. HNSCC cell lines were infected with these lentiviruses. After 48 h, the medium was removed and replaced with fresh medium containing 2 μg/mL puromycin (Solarbio, #P8230) for selection for an additional 2 weeks to enrich for cells harboring the constructs. Endogenous EZH2, MYC, UBA6, UBA1, UBE2Z, FBXW7, and RING1 were knocked down using small interfering

RNA (siRNA) (Tsingke Biotechnology Co., Ltd., Beijing, China). The sequences of the siRNAs are listed in the Table S8. The transfected HNSCC cell lines were then used for subsequent experiments.

Plasmid construction

Full-length human EZH2 or MYC cDNA was fused with an HA or Flag tag and cloned into the pcDNA-3.1 plasmid (Umine Biotechnology Co., Ltd., #BVA03). Serial deletion constructs of EZH2 (Umine Biotechnology Co., Ltd., #BW2757) or MYC (Umine Biotechnology Co., Ltd., #BW2753) were generated by PCR and subcloned into pcDNA-3.1 vector. Immunoprecipitation was conducted using anti-Flag (1:50; Cell Signaling Technology, #14793) or anti-HA (1:50; Cell Signaling Technology, #3724) to assay the interaction between Flag-MYC and HA-EZH2 in HEK293T cells.

Cell viability and proliferation assays

5×10^3 cells with 100 μL suspension per well for cell viability assay, and 2×10^3 cells with a 100 μL suspension per well for proliferation assay were plated into 96-well plates. After 12 h, the cells were treated with corresponding drugs for indicated time, with DMSO without drug served as a negative control. Cell viability was assessed using CCK8 (HANBIO, #HB-CCK-8-500T). Briefly, 10 μL of CCK8 solution was added to each well. After 2 h in culture, the optical absorbance (A) at 450 nm was detected using microplate reader (Model 680, BIO-RAD, USA). The average of the absorbance values of all wells was used to calculate the inhibition rate of proliferation: inhibition rate = (1-absorbance of experimental group/absorbance of the control group) × 100%. The half-maximal inhibitor concentration (IC50) value was determined with Prism.

Colony formation assay

800 cells/well were plated in 6-well plates. After 12 h, the cells were treated with corresponding drugs, DMSO without drug served as a negative control. The cells

were cultured for 10 days. Then Colonies were washed twice with cold PBS, fixed with 4% paraformaldehyde for 20 min, and stained with 0.1% crystal violet (Solarbio, #G1062) for 30 min. The number of colonies containing 50 cells or more cells was counted under a microscope.

Flow cytometry analysis

Cell cycle and apoptosis were analyzed using PI/RNase Staining Buffer (BD Pharmingen™, #550825) and FITC Annexin V Apoptosis Detection Kit I (BD Pharmingen™, #556547), respectively. Cells were treated with corresponding drugs for the indicated times, then harvested and washed twice with PBS. For cell cycle assay, cells were fixed with 70% ethanol overnight at 4°C and then resuspended in 500 μL of PI/RNase Staining Buffer and incubated for 15 min at room temperature (RT) in the dark before being analyzed by flow cytometry. For cell apoptosis assay, cells were resuspended in 100 μL 1X Annexin V Binding Buffer and incubated with 5 μL of FITC Annexin V and 5 μL of PI for 15 min at RT in the dark. Then 400 μL of 1X Binding Buffer was added to each tube, and the samples were analyzed by flow cytometry within 1 h. Both early apoptotic (PI-negative, Annexin V-positive) and late apoptotic (PI-positive, Annexin V-positive) cells were included in cell death determinations.

Western blot and protein half-life measurement

The cells were lysed in radio immunoprecipitation assay (RIPA) lysis buffer (GenStar, #E125-01) freshly supplemented with protease inhibitors phenylmethanesulfonyl fluoride (PMSF) (Fdbio science, #FD0100). Protein concentration was measured using bicinchoninic acid (BCA) Protein Assay Kit (GenStar, #E162-01). Equal amounts of protein lysate were loaded onto SDS-PAGE gels and transferred onto the polyvinylidene fluoride (PVDF) membrane (Millipore, #IPVH00010). Blots were blocked with 5% fat-free milk for 1 h at RT before incubating with primary antibodies overnight at 4°C. Appropriate horseradish peroxidase (HRP)-conjugated secondary antibodies were applied for 1 h at RT. Protein bands were visualized using the

ImageQuant™ LAS 500 system (GE Healthcare Life Sciences, Japan). To measure the stability and half-life of the protein, cells were treated with 100 μg/mL CHX and collected at indicated time points (0, 15, 30, 60, and 90 min), followed by Western blot of the target protein with total cell lysate. Densitometric analysis of protein abundance was determined by ImageJ software, and the protein degradation curves were generated. Antibodies used are listed in the Table S9.

Quantitative real-time PCR (qRT-PCR)

Total RNA was isolated using the TRIzol reagent (Ambion, #15596026) and converted to cDNA using the rimeScript RT reagent Kit With gDNA Eraser (Vazyme, #R323-01) according to the manufacturer's instruction. qRT-PCR was performed using the SYBR Green PCR Master Mix (Vazyme, #Q331-02). The relative expression levels of genes were calculated using the $2^{-\Delta\Delta CT}$ methods. The sequences of the primers used are listed in the Table S10.

Immunoprecipitation and ubiquitination analysis

Cells were harvested and lysed in NP-40 lysis buffer supplemented with protease inhibitors PMSF on ice for 30 min. For ubiquitination detection, cells were treated with 10 μM of the proteasome inhibitor MG132 for 6 h before being lysed. Cell lysates were centrifugated at 14,000 × g for 20 min at 4°C, and the protein concentration was determined using a BCA Protein Assay Kit. A total of 1 mg protein from lysate was incubated with anti-EZH2 (1:300; Cell Signaling Technology, #5246S), anti-MYC (1:300; Abcam, #ab32072), or anti-FBXW7 (1:200; Abcam, #ab109617) and rotated overnight at 4°C. A non-specific IgG antibody incubated with 1 mg protein lysates was used as the negative control. Next, the immune complexes were precipitated using 50 μL protein G beads (Millipore, #16-266). After rotation for 2 h at 4°C, beads with extracted proteins were washed twice with NP-40 lysis buffer, mixed with 2 × SDS loading buffer and boiled at 95°C for 10 min. The co-precipitates were analyzed by Western blot using a chemiluminescence method.

Immunohistochemistry

The protein levels of EZH2 and MYC in 20 pairs of paraffin-embedded HNSCC specimens were detected by immunohistochemistry (IHC) following standard protocols. IHC staining was evaluated by two independent pathologists. The intensity of staining in malignant cells was scored as follows to analyze the levels of protein expression[2]: + (no staining), ++ (weak staining), +++ (moderate staining), and ++++ (strong staining). An intensity score less than or equal to ++ was considered to indicate low expression, whereas a score greater than ++ was classified as high expression.

Immunofluorescence

Cells grown on a confocal dish were washed twice with cold PBS and fixed with 4% paraformaldehyde (Biosharp, #143174) at room temperature (RT) for 20 min. Next, the cells were permeabilized with 0.1% Triton X-100 (Sigma-Aldrich, #V900502) for 10 min, blocked with 5% bovine calf serum for 30 min at RT, and then incubated with primary antibodies against EZH2 (1:200; Cell Signaling Technology, #5246S) and MYC (1:200; HUABIO, #RT1149) primary antibodies at 4°C overnight. Fluorescence was developed by incubating with Alexa Fluor-labeled goat anti-rabbit IgG (1:1000; Cell Signaling Technology, #4412) or goat anti-mouse IgG antibody (1:1000; Cell Signaling Technology, #8890) for 1 h at RT. The nucleus was counterstained with 4′6-diamidino-2-phenylindole (DAPI) dye (Cell Signaling Technology, #4083S). Fluorescence images were obtained using a laser confocal fluorescence microscope (Olympus, FV3000, Japan).

Xenograft models

Cancer cells stably transfected with EZH2 vector or control vector (5×10^6 cells in 0.1 mL PBS) were subcutaneously injected into the right dorsal flanks of 4-week-old female Balb/c nude mice (SPF (Beijing) Biotechnology Co., Ltd.). Twelve days later, when tumor size reached about 100 to 150 mm^3, the mice were administered an intraperitoneal injection of PBS, EPZ-6438 (50 mg/kg) or squamocin (0.4 mg/kg)

every three days. Tumor volume (mm^3) was determined by caliper, and calculated every three days throughout the experiment according to the formula (length × $width^2$) / 2, where width and length represent the perpendicular and largest tumor diameter, respectively. At the endpoint, tumors were harvested and weighed. The tumor inhibition rate (TIR%) was calculated as follows: TIR% = (1−Wt/Wn) × 100%, where Wn is the average tumor weight of the negative control group, and Wt is the average tumor weight of the mice in the test group. The excised tissues were fixed in 10% neutral-buffered formalin and used for histologic examination. Animal care and experiments were performed in strict accordance with the "Principles for the Utilization and Care of Vertebrate Animals" and "Guide for the Care and Use of Laboratory Animals" and were approved by the Animal Care and Use Committee of the Southern Medical University (SMU-L2021121).

Toxicology experiments

The median lethal dose (LD50) following rapid rapid tail vein injection over seven days was assessed to determine the optimal dosage of squamocin and rotenone in Wister rats. The lethal dose of squamocin and rotenone were found to be 400 μg/mL and 100 μg/mL, respectively. Subsequently, one-fifth of the median lethal dose was selected as the highest dose for the experiment. Wister rats were intravenous injected with squamocin at high, medium, and low doses of 80 μg/kg, 32 μg/kg, or 12.8 μg/kg, respectively, and rotenone at a high dose of 20 μg/kg daily for a week. At the end point, the blood levels of alanine transaminase (ALT), aspartate transaminase (AST), urea (UREA), and creatinine (CREA) were assessed by biochemical analyses. The excised tissues were fixed in 10% neutral-buffered formalin and used for histologic examination.

ChIP-coupled quantitative PCR analysis

Immunoprecipitation assays were performed using the ChIP assay kit (Cell Signaling Technology, #9003). SCC25 cells were treated with formaldehyde for cross-linking, followed by ChIP with H3K27me3 antibody (1:50; Cell Signaling Technology, #9733)

or rabbit IgG (1:50; Cell Signaling Technology, #2729). DNA fragments were purified and analyzed by quantitative PCR. Primers for UNC5B ChIP-qPCR: Forward, GAGTCCCAGTCCACCTGTTG; Reverse, CAGAGGTGAGGTGAAGGCAG.

RNA-seq and data analysis

Total RNA was first purified using TRIzol Reagent and then assessed by 1% agarose gel electrophoresis and the Bioanalyzer 2100 system. Sequencing libraries were constructed using the NEBNext Ultra II TM RNA Library Prep Kit for Illumina. Clean reads were generated by removing low-quality reads and then aligned to the Human Genome Assembly GRCh38/hg38 using Hisat2 v2.0.5 (https://github.com/ DaehwanKimLab/hisat2). FeatureCounts v1.5.0-p3 was used to count the reads mapped to each gene, and then the fragments per kilobase of sequence per million mapped reads (FPKM) of each gene were calculated based on the length of the gene and read counts mapped to the gene. Differential gene expression analysis was performed using the R package DESeq2 v1.16.1. Differentially expressed genes (DEGs) were defined as follows unless otherwise indicated: Upregulated DEGs, log2 (fold change) > 0, FDR < 0.05; downregulated DEGs, log2 (fold change) < 0, FDR < 0.05. DAVID web server (https://david.ncifcrf.gov/summary.jsp) was adopted to conduct gene ontology enrichment analysis for the commonly upregulated or downregulated DEGs between SCC15 and SCC25 cells treated with squamocin (10 μg/mL) for 24 h. Gene set enrichment analysis (GSEA) was conducted with the GSEA software (https://www.broadinstitute.org/gsea) by exploring the Molecular Signatures Database (https://www.broadinstitute.org/gsea/msigdb/annotate.jsp).

Analysis of public HNSCC datasets

The mRNA expression data and corresponding clinical features of HNSCC patients were downloaded from TCGA (https://portal.gdc.cancer.gov/). There are 468 HNSCC patients in the TCGA dataset with a survival time of at least 30 days. In Figure 6J, optimal cutoff levels for survival analyses were determined using survminer package (https://CRAN.R-project.org/ package=survminer). In Figure 6K, median values were

used as the cutoff. Survival analyses were performed using an R program with survival (https://cran.r-project.org/web/packages/survival/ index.html).

Mitochondrial respiratory Complex I activity

The activity of the mitochondrial respiratory Complex I was assessed using a colorimetric method with commercial kits (Abbkine, #KTB1850) and a Cytation5 instrument (BioTek, USA) following manufacturer instructions.

ATP assay

Intracellular adenosine triphosphate (ATP) concentration was analyzed using an ATP chemiluminescence assay Kit (Elabscience, #E-BC-F002) according to the manufacturer's instructions. Chemiluminescent signals were detected using a Cytation5 instrument (BioTek, USA)

Determination of ROS levels

Intracellular reactive oxygen species (ROS) levels were determined using a ROS detection assay kit (Elabscience, #E-BC-K138-F) following the manufacturer's protocol. The treated cells were subjected to fluorescence analysis using a Cytation5 instrument (BioTek, USA).

Molecular docking

The molecular structure of squamocin was retrieved and downloaded from PubChem (https://pubchem.ncbi.nlm.nih.gov/). Target fishing analysis was conducted using the SwissTargetPrediction website (http://swisstargetprediction.ch/). The computational docking model was constructed using the AutoDock vina program. The protein structures of HSP90α (PDB: 2QG2) and other proteins were obtained from the Protein Data Bank (PDB, https://www.rcsb.org/).

Differential scanning fluorimetry assay (DSF)

DSF experiments were conducted using Bio-Rad CFX ConnectTM RealTime System.

First, 13.7 μL of Tris buffers (50 mM), 2 μL of protein (9 μM) and 0.5 μL of compound were added per well, followed by the addition of 1.8 μL of SYPRO Orange mixture (50×). Thermal denaturation was achieved using a temperature ramp from 25°C to 100°C at a rate of 0.5°C per minute.

Surface plasmon resonance (SPR)

The binding affinities of squamocin for its target proteins HSP90α were assayed using a SPR-based Biacore 8 K instrument (GE Healthcare). The CM5 sensor chip was used to immobilize 18,000 resonance units (RU) of the target protein to the sensor surface via a standard amine coupling reaction at 25°C in a PBS running buffer. Gradient concentrations of squamocin containing 5% DMSO were injected into the channels to evaluate the binding affinity. The dissociation constants (K_D values) of the squamocin–HSP90α complexes were calculated using Biacore 8 K Evaluation Software. A kinetic method was employed to fit the results: Kinetics $Chi^2(RU^2)$ =1.93; ka (1/Ms) =1561.36; kd (1/s) =0.03.

Cellular thermal shift assay (CETSA)

293T cells were transfected with plasmids encoding HSP90α WT (wild type)/HSP90α Mutant (N51A, G97A, F138K, Y139R) pcDNA3.1-3×Flag-C using Lipo3000 for 48 h. After incubating with squamocin for 6 h, the cells were divided into equal volumes in PCR tubes and incubated at a series of temperatures from 40°C to 64°C, with a gradient of 3°C for 3 min. After being frozen by using liquid nitrogen and thawed on ice for 3 times, the supernatant was collected for the subsequent Western blot analysis.

Patient-derived xenograft mouse model

The tumor tissues for preparing PDX mouse model were originally resected from CRC patient with signed consent for the use in pre-clinical research. Briefly, under anesthesia and sterile conditions, a small incision on the skin was made in the skin of the flank area of NSG mice[3]. Next, the harvested tumor fragment was placed subcutaneously, and the wound was closed with stitches. This was considered as

passage 0 of the PDX mouse model. The tumor was resected when it reached the volume of 1,500 mm^3, and subsequently reimplanted into mice following the same protocol until passage 3. At this point, whole-exon sequencing confirmed that the model was stable, which guarantees growth upon reimplantation. Tumor volume (mm^3) was determined using caliper and calculated every three days throughout the experiment according to the formula (length × $width^2$) / 2.

Whole exome sequencing (WES) analysis

A total amount of 200 ng genomic DNA (gDNA) per sample was used for the DNA sample preparations. Sequencing libraries were generated using QIAprep EZ DNA Library Kit, following the manufacturer's instructions. Briefly, the gDNA was randomly fragmented to a size of 150-250bp, then the DNA fragments were end-polished, A-tailed, and ligated with adapters for Illumina sequencing. The PCR products were purified using beads and then were amplified for 5-6 cycles. Exome capturing was done using the QIAhyb Reagent Kit. The library was analyzed for size distribution using an Agilent 2200 Bioanalyzer and quantified using a Qubit fluorometer. Finally, the library was sequenced using Illumina NovaSeq 6000 with paired-end 150 bp reads. Raw reads were filtered to obtain clean reads by removing sequencing adapters, short reads (length <30 bp) and low-quality reads using fastp (v0.12.4). Then FastQC was used to perform quality control. Clean reads were aligned to the reference genome using BWA-MEM2 (v2.2.0) and samtools (v1.5) with a quality threshold of (-q 30). The Genome Analysis Toolkit (GATK, v4.2.2.0) was used for variant discovery. Mosdepth (v0.2.9) was used for BAM file quality control. Control-FREEC (v11.6) was used to call copy number variations (CNVs). Lumpy (v0.2.13) and svtyper (v0.7.1) were used to analyze structural variants (SVs). Functional annotation of the detected sequence variants was performed by annovar. All softwares were used with default parameters.

Statistical analysis

Statistical analysis was conducted using IBM SPSS Statistics 26. Data are presented

as the mean ± SEM. Comparisons between two groups were assessed using a two-tailed Student's t-test, whereas statistical significance among three or more groups was calculated using two-way ANOVA. The difference in the expression of each molecule in ranked data was calculated using the Wilcoxon matched-pairs signed rank test. Survival data were plotted as Kaplan–Meier curves, and significance was estimated using log-rank test. A p-value (two-sided) < 0.05 was considered statistically significant. ns, non-significant, *p < 0.05, **p < 0.01, ***p < 0.001.

2. Figures S1 to 9

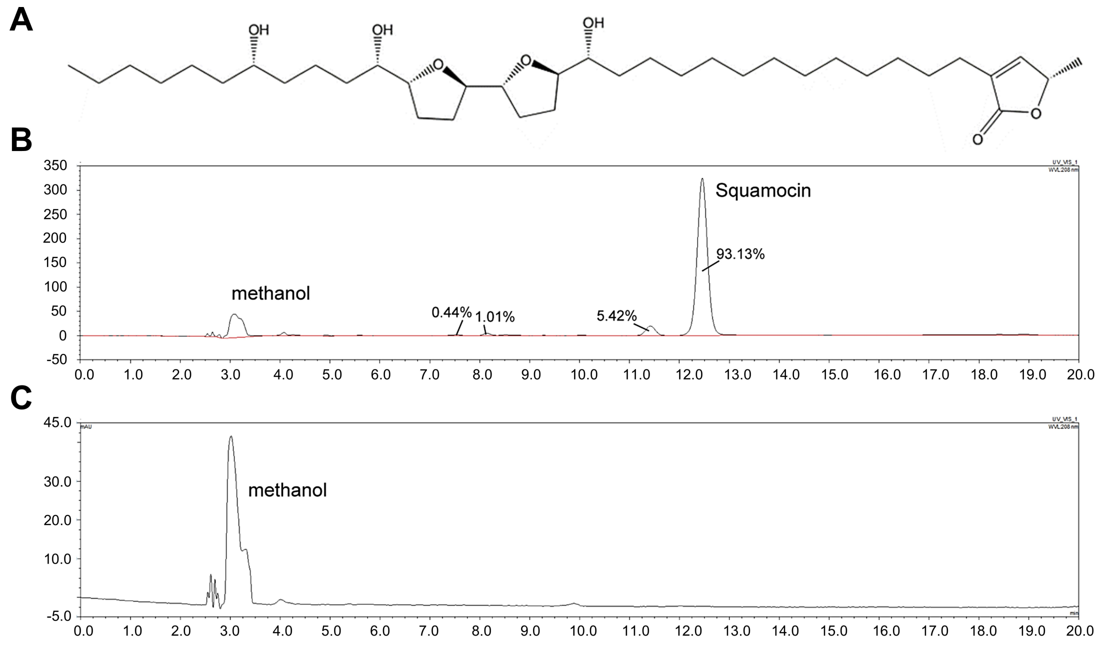

Figure S1. HPLC identification of squamocin. A) Structure of squamocin. B) HPLC chromatography of the isolated squamocin. C) HPLC chromatography of the solvent.

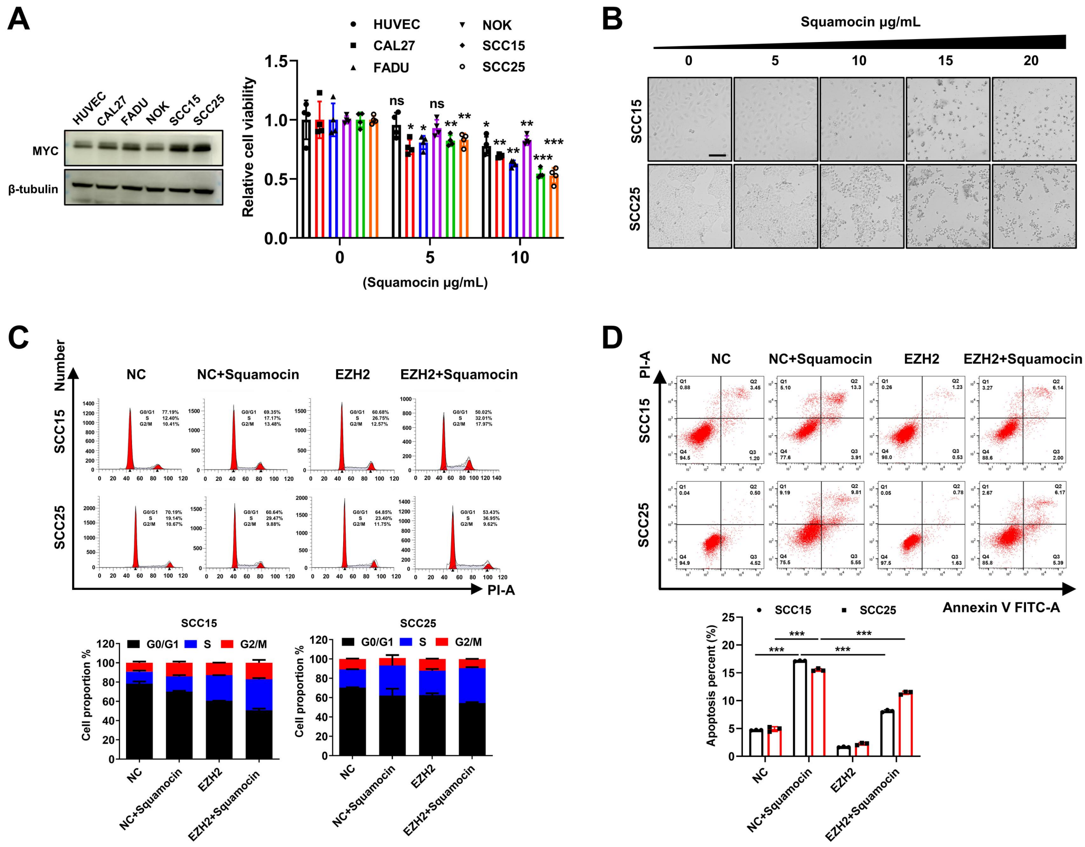

Figure S2. Squamocin represses HNSCC cell lines proliferation in an EZH2-dependent manner. A) Western blot analysis of the expression of MYC in indicated cell lines (left panel). Cells were treated with squamocin for 24 h, and cell viabilities were measured by CCK8 (right panel, mean ± SEM; n = 4, Student's t-test). B) Representative images of the cells treated with the indicated concentrations of squamocin for 24 h. Scale bars: 50 µm. C, D) Determination of the cell cycle using PI staining (C) and apoptosis using Annexin V staining (D) in EZH2-overexpressing SCC15 and SCC25 cells following treatment with 10 µg/mL squamocin for 24 h (mean ± SEM; n = 3, Student's *t*-test). *ns*: non-significant, **p* < 0.05, ***p* < 0.01, and ****p* < 0.001.

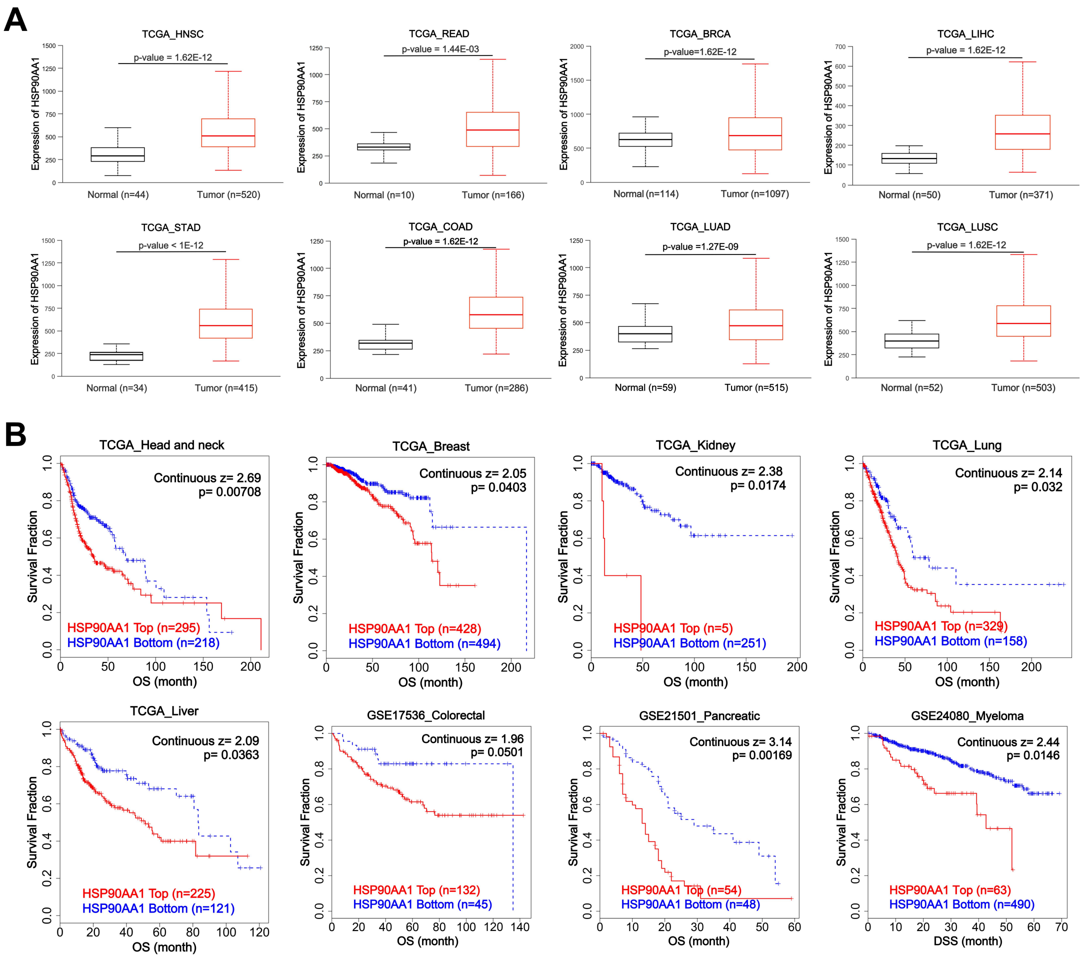

Figure S3. Expression and survival analyses of HSP90α in multiple tumor types. A) Expression of HSP90α was analyzed across multiple tumor types using TCGA database. B) Survival analysis was conducted for patients with high or low expression of HSP90α using the TCGA and GEO databases.

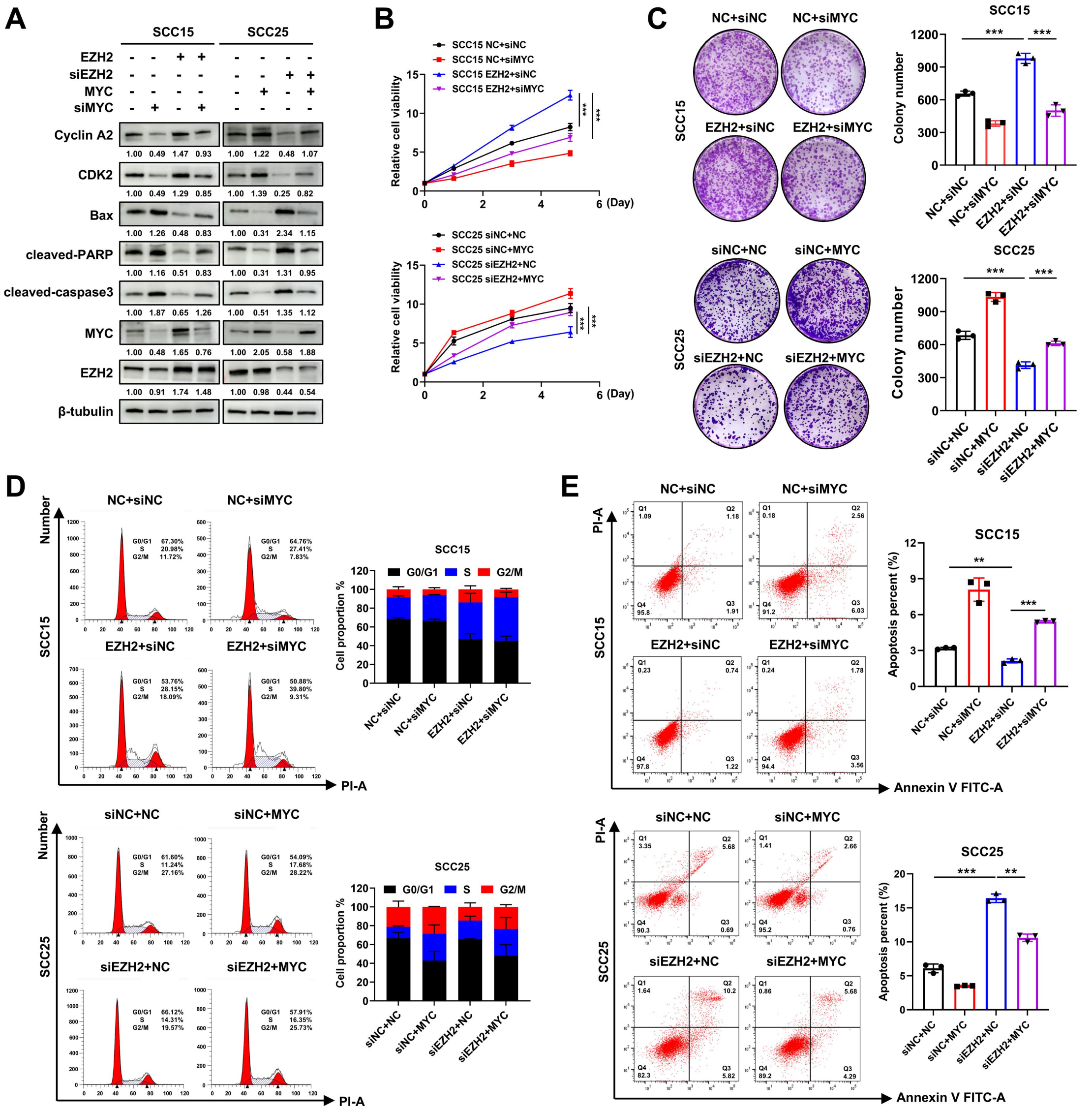

Figure S4. MYC is involved in EZH2-induced proliferation in HNSCC. A) EZH2-overexpressing SCC15 cells were transfected with MYC siRNA, and EZH2-knockdown SCC25 cells were transfected with MYC overexpression plasmids. Western blot analysis of the corresponding proteins in indicated cells after transfection for 72 h. B, C) SCC15 and SCC25 cells were transfected with indicated plasmids for 24 h for cell proliferation assays. Representative cell proliferation curves are shown (B) (mean ± SEM; n = 4, two-way ANOVA test). Representative images and quantification of clones in the indicated cells 10 days after plating (C) (mean ± SEM; n = 3, Student's t-test). D, E) Flow cytometry analysis of the cell cycle by PI staining (D) and the cell apoptosis by Annexin V staining (E) in transfected SCC15 and SCC25 cells (mean ± SEM; n = 3, Student's t-test). *$p < 0.05$, **$p < 0.01$, and ***$p <$

0.001.

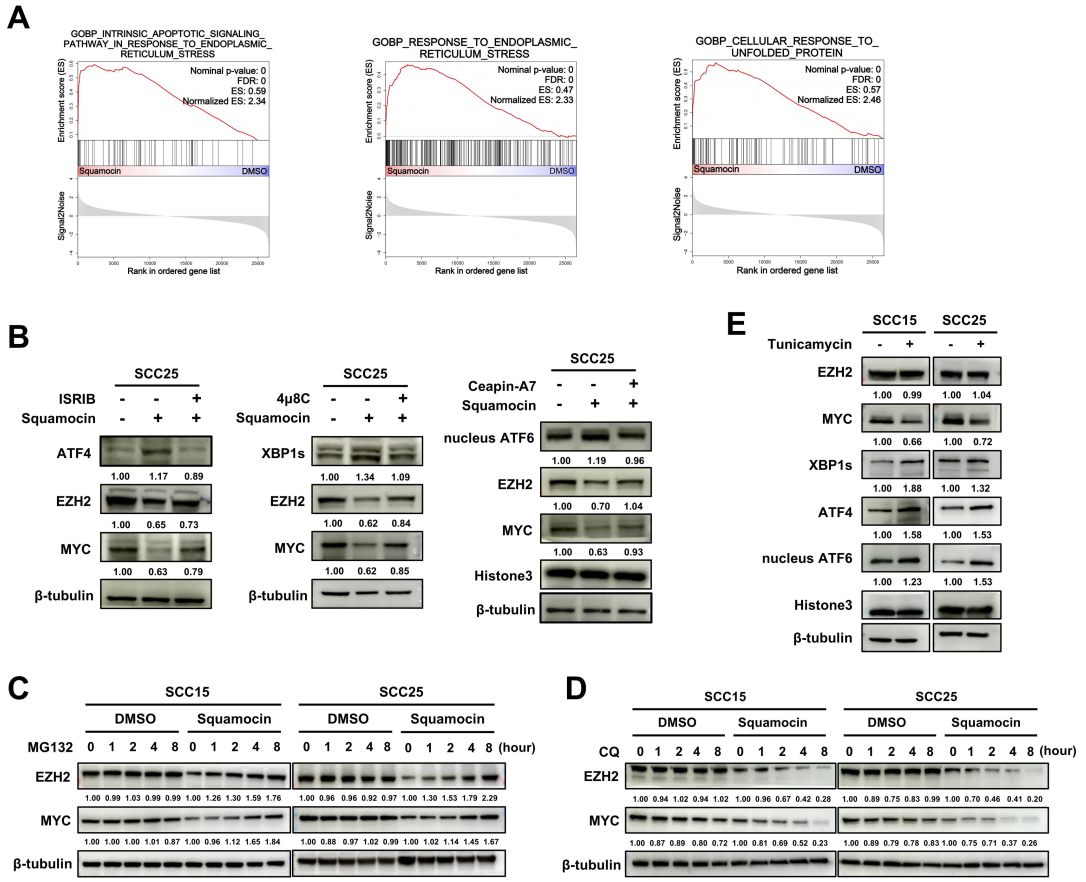

Figure S5. Squamocin activates endoplasmic reticulum (ER) stress and enhances ubiquitin-proteasome activity to degrade EZH2 and MYC. A) GSEA showing regulation of the response to ER stress and unfolded protein in SCC15 and SCC25 cells treated with squamocin. B) Western blot analysis of EZH2 and MYC in SCC25 cells treated with 10 μg/mL squamocin for 24 h and ER stress inhibitors (ISRIB: 20 μM, 4μ8C: 20 μM, CeapinA7: 20 μM) for 12 h. C, D) SCC15 and SCC25 cells were treated with DMSO or 10 μg/mL squamocin for 24 h, followed by 10 μM MG132 (C) or 10 μM CQ (D) treatment as indicated. Cell lysates were analyzed by Western blot. E) SCC15 and SCC25 cells were treated with tunicamycin (5 μg/mL) for 24 h, followed by immunoblot analysis of the indicated proteins.

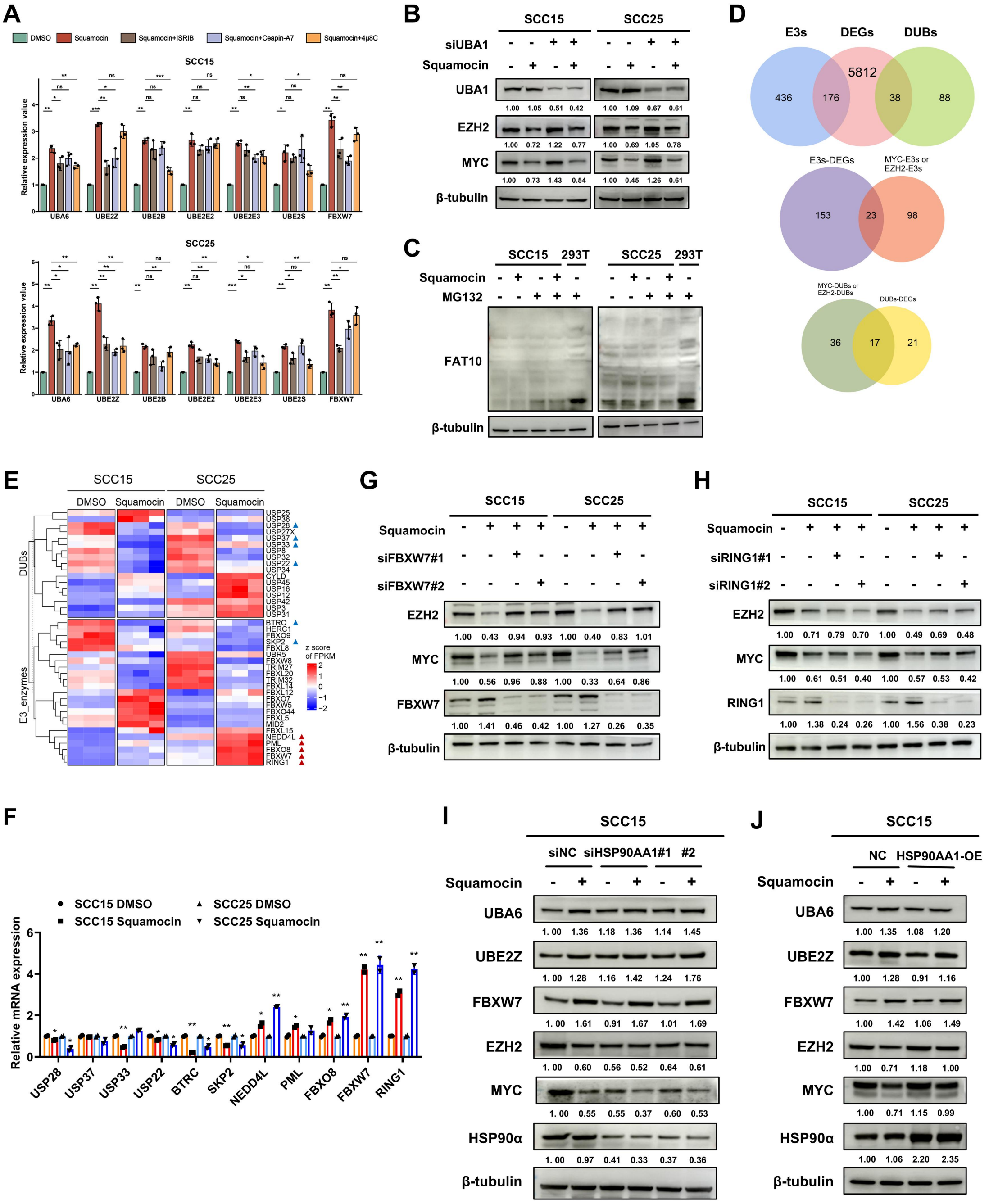

Figure S6. Screening relative ubiquitin enzymes responsible for degrading EZH2 and MYC. A) SCC15 and SCC25 cells were treated with squamocin (10 μg/mL) for 24 h and ER stress inhibitors (ISRIB: 20 μM, 4μ8C: 20 μM, CeapinA7: 20 μM) for 12 h, followed by qRT-PCR analysis of the indicated genes (mean ± SEM; n = 3, Student's t-test). B) SCC15 and SCC25 cells were transfected with siUBA1 constructs for 48 h, then treated with 10 μg/mL squamocin for 24 h, followed by immunoblot analysis of the indicated proteins. C) SCC15 and SCC25 cells were treated with 10 μg/mL squamocin for 24 h and with MG132 (10 μM) for 6 h and conducting

immunoblot analysis of FAT10. D) Venn diagram showing overlapped DEGs (FDR < 0.05) with E3s or DUBs gene set from the iUUCD 2.0 database (upper panel). Venn diagram showing the overlap of genes screened in the upper panel with E3s or DUBs interacting with EZH2 or MYC from UbiBrowser_v2 database (middle and lower panels). E) Heatmap showing 23 E3s and 17 DUBs genes screened from panel D. F) qRT-PCR was used to assess the mRNA level of indicated genes in SCC15 and SCC25 cells treated with 10 μg/mL squamocin for 24 h (mean ± SEM; n = 2, Student's *t*-test). G, H) Western blot analysis of EZH2 and MYC expression in SCC15 and SCC25 cells after transfection with two (#1 and #2) siFBXW7 (G) or siRING1 (H) for 72 h. I, J) SCC15 cells were transfected with siHSP90AA1 (I) or HSP90AA1-overexpressing (J) constructs for 48 h, then treated with 10 μg/mL squamocin for 24 h, followed by immunoblot analysis of the indicated proteins. *ns*: non-significant, *$p < 0.05$, **$p < 0.01$, and ***$p < 0.001$.

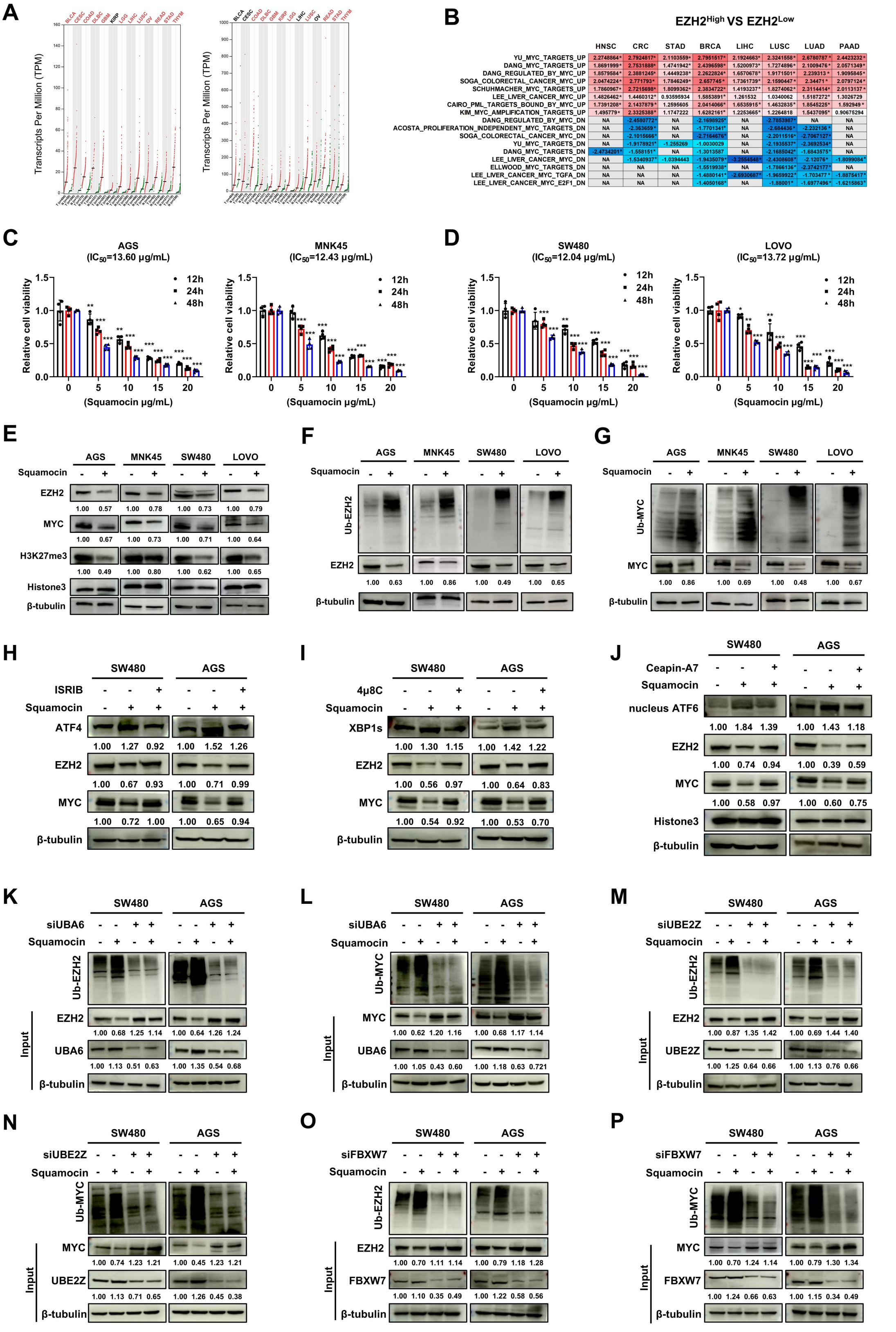

Figure S7. Squamocin effectively induces EZH2 and MYC degradation in GC and CRC cell lines through UBA6-UBE2Z-FBXW7 ubiquitin cascade. A) Analysis of EZH2 and MYC expression in pan-cancers using the TCGA database. B)

GSEA analysis of the correlation of EZH2 with MYC-target genes across multiple tumor types. C, D) GC (C) and CRC (D) cell lines were treated with squamocin at the indicated concentrations for 12, 24, and 48 h. Cell viabilities were measured by CCK8 (mean ± SEM; n = 4, Student's t-test). E) GC and CRC cell lines were treated with 10 μg/mL squamocin for 24 h, followed by immunoblot analysis of the indicated proteins. F, G) Western blot analysis of the ubiquitination of EZH2 (F) or MYC (G) in GC and CRC cell lines treated with 10 μg/mL squamocin for 24 h. H-J) Western blot analysis of EZH2 and MYC in GC and CRC cell lines treated with 10 μg/mL squamocin for 24 h, and with ER stress inhibitors ISRIB: 20 μM (H), 4μ8C: 20 μM (I) or CeapinA7: 20 μM (J) for 12 h. K-P) GC and CRC cell lines were transfected with siUBA6 (K and L), siUBE2Z (M and N) or siFBXW7 (O and P) for 48 h, followed by squamocin (10 μg/mL) treatment for 24 h. Cell lysates were immunoprecipitated with anti-EZH2 or anti-MYC and analyzed with anti-ubiquitin. *$p < 0.05$, **$p < 0.01$, and ***$p < 0.001$.

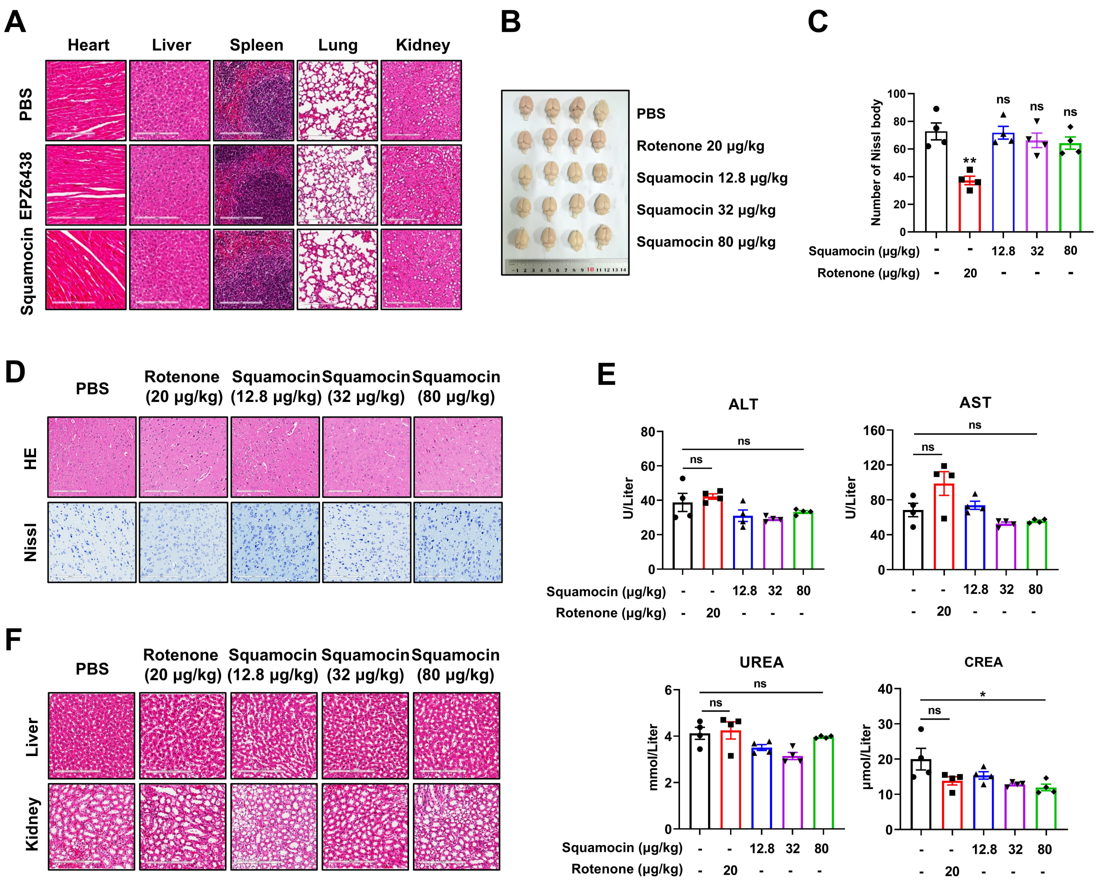

Figure S8. Toxicity analysis of squamocin *in vivo* therapy. A) Histological

morphology of the heart, liver, spleen, lung, and kidney in GC xenografts from Figure 7E was analyzed by HE staining. B) Representative images of brain tissues of Wistar rats with indicated treatment (n = 4). C, D) Quantification of Nissl bodies (C) and IHC staining of HE and Nissl (D) in the brain tissues of Wistar rats with indicated treatment. Scale bars: 200 μm. E) Levels of serum alanine transaminase (ALT), aspartate transaminase (AST), urea (UREA), and creatinine (CREA) were determined in Wistar rats with the indicated treatment (mean ± SEM; n = 4, Student's *t*-test). F) Histological morphology of the liver and kidney in Wistar rats with indicated treatment were analyzed by HE staining. *ns*: non-significant, **p* < 0.05, ***p* < 0.01, and ****p* < 0.001.

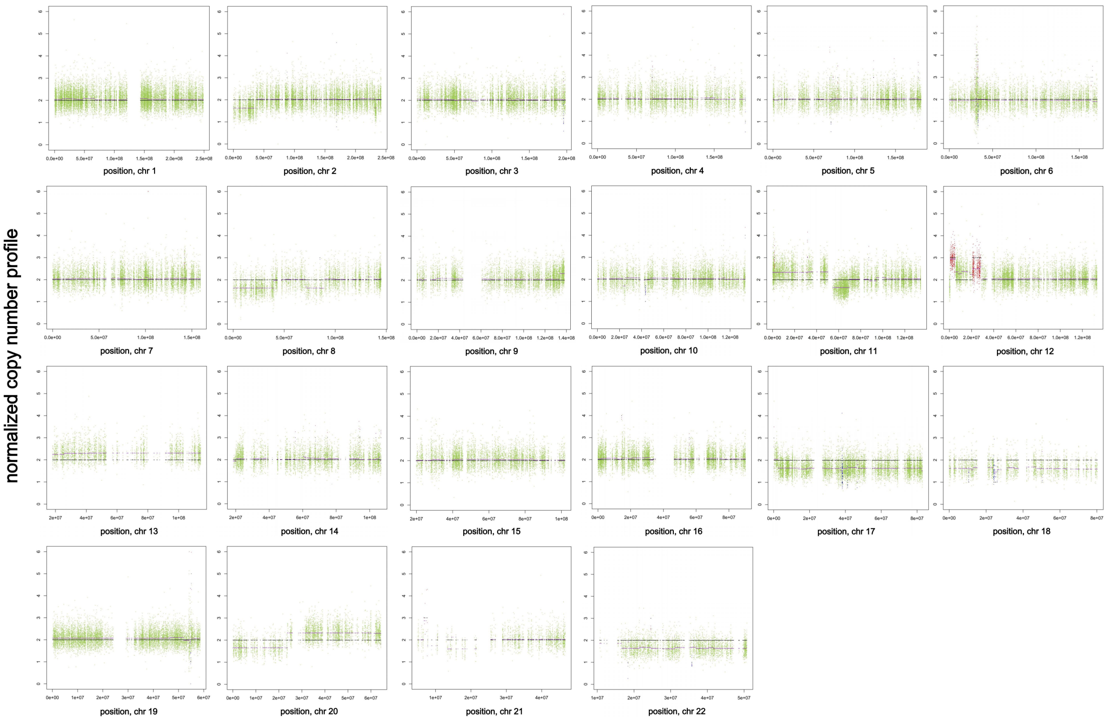

Figure S9. WES analysis of copy number variations (CNV) in PDXs and primary tumor samples. The x-axis represents the position of the chromosome, and the y-axis represents the CNV copy number. Blue indicates copy number reduction (loss), red indicates copy number increase (gain), and green copy number indicates no change in copy number. Black represents the predicted copy number, and purple represents the median copy number of the segment.

3. Tables S2, S8 to 10

Table S2. Clinicopathological characteristics and EZH2/MYC expression in 20 patients with HNSCC.

	Gender	Age	Location	Histology	TNM Stage	EZH2 Expression	MYC Expression
1	M	57	Gum	squamous cell carcinoma	T4N2M0	H	H
2	F	70	Gum	squamous cell carcinoma	T4N0M0	H	H
3	M	60	Floor of the mouth	squamous cell carcinoma	T2N1M0	H	H
4	M	52	Buccal mucosa	sarcomatoid carcinoma	T2N1M0	H	H
5	F	74	Gum	squamous cell carcinoma	T4N2M0	H	H
6	M	55	Tongue	squamous cell carcinoma	T1N0M0	H	H
7	M	75	Buccal mucosa	squamous cell carcinoma	T3N2M0	H	H
8	F	61	Gum	squamous cell carcinoma	T2N1M0	H	H
9	M	49	Tongue	squamous cell carcinoma	T3N1M0	H	L
10	M	48	Tongue	squamous cell carcinoma	T2N0M0	H	H
11	M	24	Tongue	squamous cell carcinoma	T3N0M0	L	L
12	F	58	Gum	squamous cell carcinoma	T2N2M0	L	L
13	M	58	Floor of the mouth	squamous cell carcinoma	T2N1M0	L	L
14	M	48	Buccal mucosa	squamous cell carcinoma	T3N0M0	H	H
15	M	36	Tongue	squamous cell carcinoma	T3N1M0	H	L
16	M	41	Tongue	squamous cell carcinoma	T3N0M0	H	H
17	M	42	Tongue	squamous cell carcinoma	T2N0M0	H	H
18	M	28	Tongue	squamous cell carcinoma	T2N0M0	H	L
19	F	68	Tongue	squamous cell carcinoma	T3N0M0	H	L
20	M	56	Tongue	squamous cell carcinoma	T2N0M0	H	L

Abbreviation: M: Male, F: Female; H: High; L: Low

Table S8. Small interfering RNA (siRNA) sequences

siRNA (5′-3′)	Sense	Antisense
siEZH2#1	CAGAAACAGCUCUAGACAATT	UUGUCUAGAGCUGUUUCUGTT
siEZH2#2	CAAAGAAUCUAGCAUCAUATT	UAUGAUGCUAGAUUCUUUGTT
siMYC	CGAUGUUGUUUCUGUGGAATT	UUCCACAGAAACAACAUCGTT
siUBA6	CAUUGCAGAAGAAGAUCAATT	UUGAUCUUCUUCUGCAAUGTT
siUBA1	CCUCCAACUUCUCCGACUATT	UAGUCGGAGAAGUUGGAGGTT
siUBE2Z	GGGAUAUCAUGUCCAUUUATT	UAAAUGGACAUGAUAUCCCTT
siFBXW7#1	GUCUCAGAAUAUACAAGUATT	UACUUGUAUAUUCUGAGACTT
siFBXW7#2	GGUUCUGAUGACAACACUUTT	AAGUGUUGUCAUCAGAACCTT
siRING1#1	GAUCAGACCACAACGAUGATT	UCAUCGUUGUGGUCUGAUCTT
siRING1#2	GAAAGAAGCUGGUGUCCAATT	UUGGACACCAGCUUCUUUCTT
siHSP90AA1#1	CCAGAAUGAAGGAGAACCAGA	UCUGGUUCUCCUUCAUUCUGG
siHSP90AA1#2	CGUGAGAUGUUGCAACAAA	UUUGUUGCAACAUCUCACG

Table S9. Antibodies used for immunoblotting

Antibodies	Source	Identifier
β-Tubulin	Cell Signaling Technology	Cat# 2128S
EZH2	Cell Signaling Technology	Cat# 5246S
MYC	Abcam	Cat# ab32072
Flag tag	Cell Signaling Technology	Cat# 14793
HA tag	Cell Signaling Technology	Cat# 3724
Phospho-MYC S62	Abcam	Cat# ab185656
Phospho-MYC T58	Abcam	Cat# ab185655
Cyclin A2	Cell Signaling Technology	Cat# 4656S
CDK2	Cell Signaling Technology	Cat# 2546S
Cyclin B1	Cell Signaling Technology	Cat# 12231T
Bax	Cell Signaling Technology	Cat# 5023T
Cleaved PARP	Cell Signaling Technology	Cat# 5625T
Cleaved Caspase-3	Cell Signaling Technology	Cat# 9661
H3K27me3	Abcam	Cat# ab6002
Histone3	Abcam	Cat# ab1791
Ki67	Cell Signaling Technology	Cat# 9449S
H3K27me3	Cell Signaling Technology	Cat# 9733S
MYC	HUABIO	Cat# RT1149
ATF6	Proteintech	Cat# 66563-1-Ig
XBP1s	Proteintech	Cat# 24868-1-AP

ATF4	Proteintech	Cat# 60035-1-Ig
Ubiquitin	Cell Signaling Technology	Cat# 3936
UBA6	Proteintech	Cat# 13211-1-AP
UBA1	Proteintech	Cat# 37198-1-Ig
FAT10	Proteintech	Cat# 13003-2-AP
UBE2Z	Proteintech	Cat# 16928-1-AP
FBXW7	Abcam	Cat# ab109617
RING1	Proteintech	Cat# 15037-1-AP
HSP90α	Proteintech	Cat# 60318-1-Ig
p300	Cell Signaling Technology	Cat# 54062S
RelA	Cell Signaling Technology	Cat# 8242S
Goat Anti-rabbit HRP	Fdbio science	Cat# FDR007
Goat Anti-Mouse HRP	Fdbio science	Cat# FDM007
Anti-rabbit IgG (H+L), F(ab')2 Fragment (Alexa Fluor® 488 Conjugate)	Cell Signaling Technology	Cat# 4412
Anti-mouse IgG (H+L), F(ab')2 Fragment (Alexa Fluor® 594 Conjugate)	Cell Signaling Technology	Cat# 8890

Table S10. Primer sequences used for qRT-PCR

Gene	Forward	Reverse
β-actin	CATGTACGTTGCTATCCAGGC	CTCCTTAATGTCACGCACGAT
EZH2	GTACACGGGGATAGAGAATGTGG	GGTGGGCGGCTTTCTTTATCA
MYC	GTCAAGAGGCGAACACACAAC	TTGGACGGACAGGATGTATGC
FUT3	CTGTCCCGCTGTTCAGAGATG	AGGCGTGACTTAGGGTTGGA
TERT	TCACGGAGACCACGTTTCAAA	TTCAAGTGCTGTCTGATTCCAAT
TP53	CAGCACATGACGGAGGTTGT	TCATCCAAATACTCCACACGC
UBE2C	GACCTGAGGTATAAGCTCTCGC	CAGGGCAGACCACTTTTCCTT
ODC1	GTTTTGCGGATTGCCACTGAT	GCTCTTTCGCCCGTTCCAA
BMI1	CCACCTGATGTGTGTGCTTTG	TTCAGTAGTGGTCTGGTCTTGT
PCNA	GCGTGAACCTCACCAGTATGT	TCTTCGGCCCTTAGTGTAATGAT
BCL2	AAAAATACAACATCACAGAGGAAGT	TTCTGGTGTTTCCCCCTTGG
CCND1	GCTGCGAAGTGGAAACCATC	CCTCCTTCTGCACACATTTGAA
CDC25A	GTGAAGGCGCTATTTGGCG	TGGTTGCTCATAATCACTGCC
UBA6	AGAGGTACGTTCTTGGAGACAC	TGGTTCCTAGATCCCATGCTT
UBE2Z	CAGGCCCATTTGACACTCCTT	GTCATCAGTTTGACCCGAGGT
UBE2B	TCTCTGCTGGATGAACCGAAT	ACAATGGCCGAAACTCTTTTCTC
UBE2E2	GTGAAAGTGTTCAGCAAGAACCA	GTTGACAATTTAGCAGCGGTTTT
UBE2E3	TGGAGTCCCGCTTTGACTATT	ATCCTGTCGTGTTCTGCTCTG

UBE2S	CCGACACGTACTGCTGACC	GCCGCATACTCCTCGTAGTTC
FBXW7	ACTGGGCTTGTACCATGTTCA	TGAGGTCCCCAAAAGTTGTTG
USP28	CCCTGGATCTATTAAAGGGAGCA	GCTGGAATGCGTCCTCTAGC
USP37	CCAGTGGAGCGAAACAAAGC	CCTCTGCATCCTTACTTGGTACT
USP33	AAAATCCCTTGGTACTTGTCAGG	TCGAAGAGTGGTAAGGTTCACA
USP22	CCATTGATCTGATGTACGGAGG	TCCTTGGCGATTATTTCCATGTC
BTRC	ACCAACATGGGCACATAAACTC	TGGCATCCAGGTATGACAGAAT
SKP2	ATGCCCCAATCTTGTCCATCT	CACCGACTGAGTGATAGGTGT
NEDD4L	GACATGGAGCATGGATGGGAA	GTTCGGCCTAAATTGTCCACT
PML	CGCCCTGGATAACGTCTTTTT	CTCGCACTCAAAGCACCAGA
FBXO8	AGCAAGGCTACCTCACCAGA	TCCTTCCTGTTCTTTCGATTTCC
RING1	AGAATGCCAGCAAAACGTGG	AGATAGGGCACATGAGTTCTGA
E2F1	CATCCCAGGAGGTCACTTCTG	GACAACAGCGGTTCTTGCTC

4. Supplementary References

[1] Y. Miao, X. Xu, F. Yuan, Y. Shi, Y. Chen, J. Chen, X. Li, *Nat Prod Res* **2016**, *30* (11), 1273, https://doi.org/10.1080/14786419.2015.1055490.

[2] B. Wang, L. Zhang, L. Zhao, R. Zhou, Y. Ding, G. Li, L. Zhao, *Cell Commun Signal* **2017**, *15* (1), 21, https://doi.org/10.1186/s12964-017-0179-9.

[3] G. J. Yoshida, *J Hematol Oncol* **2020**, *13* (1), 4, https://doi.org/10.1186/s13045-019-0829-z.
